# Findings from interviews with pilots on spatial disorientation: training, temporal dynamics and countermeasures

**DOI:** 10.3389/fphys.2025.1620737

**Published:** 2026-02-10

**Authors:** J. B. Dixon, T. K. Clark, T. C. Endsley

**Affiliations:** 1 The Charles Stark Draper Laboratory, Inc., Cambridge, MA, United States; 2 The University of Colorado, Boulder, Boulder, CO, United States

**Keywords:** spatial disorientation, aviation, mixed methods, thematic analysis, countermeasures

## Abstract

**Introduction:**

Spatial disorientation (SD) remains a prevalent issue in aviation, accounting for a disproportionate amount of Class A mishaps and fatalities. The current literature lacks specificity necessary to capture aspects of SD that may be essential in developing methods for detection and mitigation. This investigation focuses on the temporal progression of SD events in flight, elements of SD training facilitating recognition, recovery or avoidance of SD events, and considerations for SD countermeasures.

**Methods:**

We developed a mixed-methods research study involving a questionnaire followed by a semi-structured interview to evaluate perception of spatial disorientation within a pilot population. Thirteen pilots participated with a mean age was 60 years (range: 35–76), and a median of 6,000 (range: 317–19050 h) flight hours.

**Results:**

Pilots reported common chronological stages of recognizing and responding to SD events defined as a habitual reaction to direct attention to instrument displays, an analytical investigation of the true aircraft state, followed by a calculated control response if warranted. Pilots estimated that the typical duration an SD event may go unrecognized is on the order of 1–15 s. Estimates for the durations of the various stages of response suggest events last on the order of 1’s to 10’s of seconds. The psychological and physiological recovery associated with experiencing an SD event often persists for 10’s of seconds to minutes following recognition and recovery of the aircraft. SD-specific training was said to have positive impacts on understanding conditions where SD is likely to occur, and may reduce the duration it goes unrecognized. However, it does not appear to have much direct influence on recovery *during* an SD event.

**Discussion:**

Pilots suggest that real-time detection and pilot aiding systems would be acceptable additions to the flight deck, with caveats of signal detection performance, maintaining anonymity, and intended use of data collected. Through rich interview reports, we built a model of the temporal dynamics of SD events. This model can serve as a tool for improving specific aspects of SD training, developing computational SD detection methods, and designing targeted countermeasure systems for avoiding and/or mitigating the impacts of SD on pilot safety and performance.

## 1 Introduction

### 1.1 Spatial disorientation in aviation

Humans perceive their orientation with a multitude of sensory systems (Young, 2003). Within an aviation environment, pilots rely primarily on visual, vestibular and somatosensory cues to determine their orientation relative to Earth’s surface, and to maintain safe control of the aircraft. However, during mission-related distractions or deprivation of typical stimuli, a veridical perception of orientation is challenged (Previc and Ercoline, 2004). These difficulties, in addition to environmental factors, lead to spatial disorientation (SD) events in pilots controlling aircraft. The classic definition of SD attributed to Benson (Benson et al., 1999) is a human’s “*failure to correctly perceive attitude, position, and motion [of the aircraft]*”. These misperceptions commonly lead to inappropriate control inputs (or lack of appropriate control inputs), which when operating in the limited flight envelopes of aerospace vehicles, can lead to controlled flight into terrain (CFIT) and other accidents or mishaps, sometimes in a matter of seconds (Newman et al., 2014).

SD is a leading contributor to Class A mishaps (>$1 million in damage, or injury resulting in fatality or total disability) (Braithwaite et al., 1998; Cheung et al., 1995; Gaydos et al., 2012; Gibb et al., 2011; O’Neil et al., 2017; Poisson and Miller, 2014; Stott, 2013), and even with wide inclusion of SD-specific training (Bles, 2008), SD-related accident rates have not trended downward over the last 4 decades (O’Neil et al., 2017). Moreover, SD has a disproportionate fatality rate compared with other causes of mishaps. Over 2 decades within the USAF, it was estimated that SD-related events were 2.85 times more likely to be fatal than all other Class A mishaps, and had greater than a 60% fatality rate (Poisson and Miller, 2014). Recent surveys show that SD remains a serious obstacle in ensuring flight safety, even for aviators with SD training (Pennings et al., 2020).

SD episodes are commonly classified into various phases/types: unrecognized (Type I), recognized (Type II), and incapacitated (Type III) (Previc and Ercoline, 2004) which can be thought of as a specific subset of recognized SD. In general, most events involve some duration of Type I SD before the pilot can become aware of the misperception. It is estimated 85% of SD-related accidents are attributable to Type I SD (Stott, 2013), indicating the pilot did not recognize the presence of SD or recognized too late to correct the attitude of the aircraft. Additionally, researchers have defined various SD “illusions” (Previc and Ercoline, 2004) that encompass specific misperceptions, or series of misperceptions, that lead to common experiences. For example, many aviators experience an incorrect perception of the gravitational vector when flying above a sloped cloud deck due to interpreting it as the true horizon. It is important to note, however, that there can be substantial variability in the inertial and environmental stimuli that lead to the “same” illusion. While it has proven useful to characterize common SD experiences for the purpose of tracking SD incidence over time, the restricted specificity of grouped experiences may be limiting in developing effective defenses against SD.

### 1.2 Defenses against spatial disorientation

Much of the research aimed at preventing SD and the resulting accidents has focused on improvements to pilot training during the education phase (Bles, 2008), or possible instrumentation solutions aimed at maintaining pilot situational awareness (Paillard et al., 2014). However, SD training is the only countermeasure (CM) that has matured to broad operational use. Training includes a mix of classroom, simulator and in-flight demonstrations on the causes and consequences of SD, as well as practice performing general upset, and attitude recovery (Bles, 2008). Based on subjective reports, using a standardized survey (Braithwaite, 2002), Pennings, Oprins (Pennings et al., 2020) found there is a high appreciation for SD training that was also positively correlated with the amount of training received. However, due to the anonymous and predefined nature of the standardized postal questionnaire, it remains difficult to draw conclusions, for example, on the direct indications of positive transfer of training. Older surveys explored the impact of training in less detail, but made similar conclusions to the potential benefits of SD training on recognition of SD events in flight (Holmes et al., 2003a; Matthews et al., 2002). Taken with the more recent statistics that SD accidents are not decreasing (Pennings et al., 2020; Lewkowicz and Biernacki, 2020), this indicates that the benefit of SD training (with current resources) in terms of mitigating SD has been realized, and remains insufficient as a standalone countermeasure.

Various instrument solutions have been proposed as early as the mid-90s, with some seeing in-flight demonstrations (Malcolm, 1984; Raj and Braithwaite, 1999). Approaches aimed at enhancing or providing alternative sensory information regarding aircraft spatial orientation include auditory (Brill et al., 2016), visual (Malcolm, 1984; Eriksson, 2009; Previc, 2012), somatosensory (Rupert, 2000; Van Erp et al., 2006) or “multisensory” displays involving a mix of individual instruments (Albery, 2007; Wickens et al., 2007). As Paillard, Quarck (Paillard et al., 2014) notes in their review of potential sensorial CMs, those focused on the vestibular sensory cues are limited to pilot training through simulator experience or galvanic vestibular stimulation exposure, neither of which have been proposed as real-time solutions.

The principal concern with adding instrument CMs is the risk of increasing pilot mental workload, and elevating the opportunity for distraction. Balancing the interdependency of workload and situational awareness is central in the design of CMs, particularly in the high-demand context of flying aircraft (Wickens, 2002). It is paramount that the available attentional resources of the pilot are demanded by a CM *only when* needed, in order to take advantage of potential benefits of instrument solutions without creating additional mental workload, distraction, and the associated risk. Thus, the ability to monitor the risk of a pilot experiencing SD is needed to act as a real-time trigger for directed CMs.

Several metrics relating to detecting SD events have been proposed. While there may be physiological correlates of Type II SD (Tornero Aguilera et al., 2020), they are unlikely to exist during the precipitating phase of unrecognized SD [see eitology of SD in Cheung (2013)]. Some researchers have also suggested control stick inputs may serve as an indicator of SD (Roscoe, 1997), however, utilizing such parameters requires an expectation of what the “correct” or “reasonable” signals would be over time. Although that may exist for some situations, such as when on a defined landing approach, it typically does not exist for real-time, human-in-the-loop, operational environments where the pilot is free to act as they see fit. It is unsurprising, therefore, that most efforts towards developing a detection method have focused on leveraging computational models of human orientation perception (Newman et al., 2014; Dixon et al., 2019; Groen et al., 2016; Kynor, 2002; Onur et al., 2014; Small et al., 2006; Small et al., 2011) that do not necessarily need input from the pilot or predefined expectations. Most of these modeling attempts have been applicable only to *post hoc* analysis of accident flight data as they required substantial subject matter expert (SME) interpretation of the incomplete flight data sets. In summary, all hypothesized detection methods have seen limited or halted progress due to the lack of knowledge and data surrounding the real-time dynamics of how SD is perceived and experienced in-flight, and how SD training as a CM directly influences SD experiences.

### 1.3 Gaps in spatial disorientation defense research

Much of the data that has been collected regarding SD experiences comes from survey investigations designed to quantify aspects of SD, such as frequency of events, their severity and trends over time. The motivation surrounding these studies has been to provide a more thorough understanding of the impacts of SD beyond mishap analyses, and assess any influence of SD-training on recognition and reporting. Unfortunately, early studies (Braithwaite et al., 1998; Cheung et al., 1995; Collins and Harrison, 1995; Durnford et al., 1996; Kuipers et al., 1990; Navathe and Singh, 1994; Sipes and Lessard, 2000; Tormes and Guedry, 1974) had many inconsistencies in survey design and analysis that make it difficult to draw direct comparisons or highlight trends over the years. This motivated the Air Standardization Coordination Committee, consisting of five nations’ air forces, as well as the US Navy, to develop the WP61 postal survey questionnaire to provide investigators with a standardized resource for assessing these trends (Braithwaite, 2002).

At least five research efforts utilizing the WP61 SD postal survey have been conducted since its creation in 2000. The initial two studies (2002-03) from the United States Air Force (USAF) (Matthews et al., 2002) and the UK military (Holmes et al., 2003a) show that SD remained a widespread problem even after the broad induction of SD training over the last few decades (Bles, 2008). Specifically, results show that while the most experienced illusions may differ between aircraft type, aviators of all aircraft are susceptible to SD. For some of the most commonly experienced illusions–the leans, loss of horizon, undetected drift and distraction/task saturation–there was between 55% and 95% of all respondents for any aircraft type that reported having experienced the event. Moreover, pilots reported experiencing an average of 10.6 of the 34 presented SD illusions at least once in their careers (Matthews et al., 2002). These initial studies highlighted the pervasiveness of SD despite a training countermeasure, as well as demonstrated the utility of employing a common tool for quantifying and researching SD.

Other independent efforts have included studies conducted by researchers of the Czech Republic’s Air Force (Boril et al., 2018), the Military Institute of Aviation Medicine in Poland (Lewkowicz and Biernacki, 2020), and TNO and the Royal Netherlands Air Force (Pennings et al., 2020). The results of all three studies align closely with the initial findings from Matthews et al. (2002) and Holmes et al. (2003a): the majority of aviators will experience multiple SD events throughout their career and incidence can be tied to aircraft type, SD illusion type, and amount of SD-training experience. In the case of Pennings, Oprins (Pennings et al., 2020), 7/10 of the most commonly experienced illusions aligned with Holmes et al.’s findings, and the majority of participants had experienced them across all aircraft types. Additionally, the studies partially corroborate previous findings that the amount of SD training is correlated with the number of reported events, indicating training is helpful for the awareness needed to recognize SD in-flight.

This body of literature is helpful for mapping what kinds of SD experiences should receive dedicated attention, differences between pilot and aircraft populations, trends over time, and the broad influence of SD training on those metrics. However, these questionnaire-based approaches are limited in that they: (1) ask only predefined questions, preventing any follow-up queries, (2) yield only binary or Likert-style responses, restricting the richness of the dataset, and (3) yield only summary outcomes across the survey population (e.g., average rating of appreciation of SD training). This limited specificity does not facilitate capturing nuanced aspects of SD experience that we suggest may be essential in developing useful methods of measuring and mitigating SD, and refining existing defenses such as SD training. Some of these aspects include:

1. The temporal progression and variability of experience over entire SD events (e.g., what range of durations do unintended attitudes occur within that lead to “leans” illusions before recognition?)
2. The positive transfer of training on recognition or avoidance of SD (e.g., does motion-based simulator training lead to shorter durations of unrecognized Type I SD?)
3. How CMs should function to ensure a positive impact on flight safety and performance (e.g., what levels of signal detection performance for a real-time SD detection system are required from a pilot’s perspective to make intervention useful?).

We developed a semi-structured interview to follow up with respondents of an initial survey [adapted from the WP61 (Braithwaite, 2002)] in order to address these unknown gaps in knowledge and facilitate reaching unanticipated conclusions. Specifically, we sought to collect a rich dataset allowing researchers to (1) analyze and improve the benefits of training as a passive CM to SD, (2) identify and inform possible *real-time* detection strategies for unrecognized SD by understanding the temporal progression of SD experiences, and (3) design effective active CMs in hopes of helping prevent future SD-related incidents.

## 2 Methods

### 2.1 Mixed-methods design

Mixed methods is a rapidly growing and evolving research paradigm that employs multiple, distinct methods for conducting research, collecting data, and/or exploring related philosophical ideas (Creswell and Creswell, 2018; Johnson et al., 2016). There are many definitions of mixed methods from different leaders in the field, but for the purposes of this study we used the definition that “*mixed methods research is a research design (or methodology) in which the research collects, analyzes, and mixes (integrates or connects) both quantitative and qualitative data in a single study or a multiphase program of inquiry*” [quote from 46, Table 1]. In the case of this study, this includes a static survey analyzed quantitatively, and a semi-structured interview analyzed using thematic analysis. Thematic analysis is a standard qualitative method used to identify, analyze and report patterns (themes) within qualitative data. In this process, “codes” are assigned to critical statements from the interview by subject matter experts, and then grouped into broader patters which represent overarching ideas or concepts that constitute the main findings of the interviewers generated across respondents. This process is described in more detail in Section 2.4.

**TABLE 1:** Participant totals. Participant IDs (column 1) ordered based on Total flight hours (column 7). Total flight hours across airframes (columns 2–6) and career (column 7), and total number of illusion types (of the 33 listed in the survey) reported having been experienced “Rarely”, “Seldom” or “Occasional” for any airframe.

Participant ID	Fast-jet hrs	Rotary-wing hrs	Single-engine hrs	Multi-engine hrs	Other Hrs	Total hrs	# SD illusion types reported (N/33)
M	-	-	350	-	-	350	8
B	-	316.6	-	-	-	316.6	13
A	200	-	1800	500	-	2,500	7
L	2,362	6	-	337	100	2,805	20
J	252	-	1,551	2,211	-	4,014	14
K	3,800	-	1,400	-	-	5,200	5
C	-	-	5,977	-	-	5,977	10
E	2000	350	4,000	2020	45	8,415	11
G	2,500	-	1,300	3,500	1,200	8,500	27
I	4,550	-	613	3,937	-	9,100	22
H	5,500	10	2,500	5,300	-	13,310	16
F	-	-	20	15,780	-	15,800	9
D	4,000	-	3,000	12,000	50	19,050	5

The limitations of past surveys include a lack of specificity regarding positive transfer of training and temporal dynamics of SD events, as well as the ability to uncover previously unfound trends in the experience and handling of SD events. The methodology developed and used in this study seeks complementarity (interpretability, validity), initiation (depth of inquiry from multiple perspectives) and expansion (scope of inquiry from multiple components) of the data collected regarding the experience of spatial disorientation. To assist in the development of quantitative detection methods an open-ended semi-structured interview preceded by a survey was implemented. The survey was adapted from the standardized WP61 postal survey questionnaire used widely by SD researchers and can be seen in Supplementary Appendix A. This was used to determine the prior SD experiences of participants, and provide context for personalized interview follow-up questions. Additionally the survey served as a comparative instrument for prior pilot populations evaluated within the WP61 postal survey. In order to gather richer data associated with pilot experiences of SD, we developed a 45-item semi-structured interview Supplementary Appendix B instrument based on three main gaps:

1. The direct influence of SD training on the recognition, perception and reaction to SD episodes;
2. The temporal progression of SD as experienced in flight; and
3. Considerations from the pilot’s perspective on the intrusiveness, method of countermeasure delivery, and data anonymity of a hypothetical SD detection and pilot aiding system.

One important benefit of the rich qualitative data collected through interviews is the reduction of bias involved from the research investigators. Interview responses capture the voice of participants for experiences that can be understood in deeper context, and is based on the views of participants rather than the researcher (Creswell, 2014). Although the WP61 survey attempts to be comprehensive in the types of SD experiences pilots can report on, it is strictly limited by framing SD experiences in the context of SD illusions. Additionally, conclusions drawn from the results of Likert scales–as in the case of pilots rating their appreciation of SD training–cannot be tied to specific elements of SD training or real experiences. Surveys are also explicit in the nature of what they ask for, and cannot gather further elaboration on any open-ended responses provided. As an example, the WP61 survey includes an open-ended question of “*What was your last disorienting illusion/incident in your current aircraft type? (brief description–describe what you felt, what cause it, how you recovered)*” (Holmes et al., 2003b). Without consistent depth of context in similar responses, it is difficult to identify commonalities in the finer aspects of SD experiences. As such, this investigation aimed to address questions raised by the results of past survey studies in more depth, and potentially uncover new findings surrounding SD experience, as well as areas for future research.

### 2.2 Materials

The WP61 SD postal survey is a static, retrospective questionnaire consisting of two sections. We made adaptations to the questionnaire to better inform the follow-up interview, avoid redundant, open-ended questions, and address retrospective considerations made by the most recent formal SD survey (Pennings et al., 2020). First, participants answered questions regarding demographics and flight experience. Additional information such as flight experience on *all* airframe types, and for certifications, ratings and degrees held throughout the pilot’s career were asked to provide broader perspective to the interviewer.

Second, participants estimated the frequency per number of all sorties that they experienced particular SD events, which totaled 33 items organized into four categories: (1) visual, (2) body sense (i.e., vestibular, somatosensory), (3) miscellaneous, and (4) display. There were also an optional category of “other” for reporting SD experiences that did not conform to definitions of common SD illusions. The final portion of the WP61 survey involves open-ended questions regarding the participants *last* SD experience in flight, and details regarding the incident (aircraft, mission task, environmental conditions and indication of severity of the event). As the questions in this section were embedded within the interview script, they were not included in the initial survey for this investigation. Following the survey, participants were contacted to schedule an interview.

Semi-structured interviews were conducted by a single, trained interviewer with individual interviews lasting between 45 and 90 min. The *a priori* questions originating from the gaps in SD literature that motivated this research methodology were grouped in categories of (1) SD training and classification, (2) psychological and physiological experience throughout SD events, and (3) views on countermeasure techniques. All participants addressed all questions posed by the interviewer, and were allowed to provide as much detail and additional information as desired. The semi-structured nature of the interviews provided flexibility to skip irrelevant questions based on past responses, move fluidly between questions as different topics arose, and probe specific reports made by participants with questions that were not part of the *a priori* interview script. The audio from all interviews was recorded with informed consent for verbatim transcription and analysis.

Analysis of quantitative data collected from the survey was analyzed independently to ensure a representative sample of previous survey investigation populations. Interview reports were aggregated across participants for analysis of emergent themes. Results of these analysis stages were integrated to address open questions of past survey designs, and establish recommendations for further investigations employing this novel mixed methods design.

### 2.3 Participants

A digital questionnaire and verbal interview were administered between September 2019 and April 2020. Interviews occurred either in person or over telephone, based on participant availability and preference, by a common interviewer. Participation was voluntary and confidential. The protocol and materials were approved by the University of Colorado Boulder’s Institutional Review Board (protocol #19-0495) and all participants signed an informed consent. Participants were not compensated for their time. Study inclusion criteria included holding, or having held a pilot certification, as well as having and recalling at least one spatial disorientation event experienced in flight. Participation was not limited to aviation type (GA, Commercial and Military) or current professional status.

Thirteen of fourteen pilots that completed the survey agreed to participate in the interview, and were included in Table 1. Participants were recruited until the investigators determined thematic saturation had been achieved. Thematic saturation is defined as the point in data collection when “*the collection of data from new participants does not add substantially to the codes or themes being developed*” (Creswell, 2014). It is important to note that the scope of thematic saturation for this investigation was limited to overall information related to the three main research gaps. For example, it was not within scope to identify potential differences in training efficacy for rotary-wing vs. fast-jet pilots, or a fixed-base vs. motion-base training simulation environment. While there may exist differences related to such variables, we sought to first build a generalized common operating picture of pilot experience and opinions related to SD dynamics, training and countermeasures.

**FIGURE 1:**
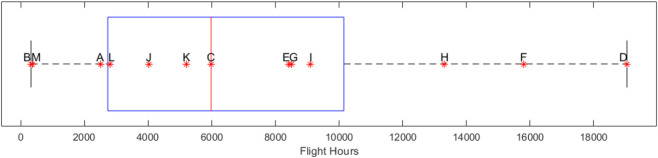
Box and Whisker plot of participant total flight hours, marked by participant identifier.

Of the 13 participants, nine were operational pilots (military, commercial), two were nonoperational pilots (GA), and two were retired. The mean age was 60 years (range: 35–76), and the median number of flight hours was 6,000 h (range: 317–19,050 h) (Figure 1). There were four pilots with rotary-wing experience with three having experience on other airframes, such that 12 of 13 total participants had fixed-wing experience. Only three pilots had experience in just one type of airframe. Additionally, participants were asked for alternative flight experiences in an “other” category. Aerospace vehicles that participants listed in this category include gliders, blimps, balloons, and spacecraft. However, there were no reported SD events associated with these experiences, so they were not taken into account in the analyses.

### 2.4 Thematic analysis

The interview transcripts were subjected to Thematic Analysis (Braun and Clarke, 2006) involving an iterative and inductive method of coding the data to produce emergent themes. The flexibility of this method is a fundamental aspect of qualitative and mixed-methods research strategies as compared to classical hypothesis testing. The limitation of an overemphasis on hypothesis testing was summarized well by Edgington (Edgington, 1967):*“Whereas research designed for testing hypotheses typically involves specifying before collecting any data the manner in which the samples of data will be taken and the procedure by which the data will be analyzed, research for the purpose of generating theories must be extremely flexible, allowing the sampling and data analysis procedures to be continuously modified along lines suggested by the data while it is being collected.”*

In the first stage of analysis, the data (consolidated across participants) was broken down from the transcripts into 679 unique statements. High-level categories were created based on common content of the interview data, and the *a priori* questions originating from gaps in literature that motivated the research efforts into SD mitigation approaches: SD training, experience, and countermeasures. This resulted in five to eight high-level categories for each of the three gaps. In the second stage, the statements were then analyzed together, and between one to five categories were developed under each high-level category for granularity. When reanalyzing the statements together within each subcategory, categories were either further refined into codes to differentiate meaning across reports, or the category became a code if it did not require additional specificity. A code is systematically identified and labeled by interesting features of the data, which can be specific words, phrases, or sentences. Ultimately, 13-30 codes were developed under each gap listed above based on diversity of the data. The coding scheme was reviewed by two co-investigators to ensure comprehensiveness, and when particular code descriptions were not sufficiently different in meaning they were combined. The unique statements were then re-coded based on the final scheme, and when a particular statement contained multiple units of meaning it was assigned multiple codes. This process resulted in 1,145 code assignments across 61 unique codes, for an average of about 19 unique statements per code. Final codes and descriptions organized by the three motivating gaps are listed in Supplementary Appendix C. Further detail and a visual representation of this coding process has been published by Osei-Amanfi (2018).

Each group of statements were analyzed individually to refine emergent themes that inspired the coding scheme (*complementarity*), and potentially discover new themes not previously realized through the ungrouped responses (*initiation, expansion*). These findings were collated across subcodes where appropriate, and the resultant themes generated by the codes are discussed here with respect to the questions motivating the study.

## 3 Results

### 3.1 Initial survey: participant population

For this investigation, the intent of the initial survey was twofold: to verify our cohort is representative of previous survey populations, and to provide context for the semi-structured interview. Our intended sample size for this interview investigation is necessarily small relative to past survey studies that collect on the order of hundreds to thousands of responses representing up to millions of flight hours. Therefore, statistical analyses commonly seen in past survey studies such as correlation tests and analysis of variances, were not conducted. While the survey results themselves do not contribute conclusions or recommendations of this investigation, we include a brief analysis of the results here to support continuity of standardized data collection and future analysis of trends over time.

#### 3.1.1 SD experiences

Table 2 summarizes the results of the survey, showing the occurrence of SD is pervasive, as found in past reports. The data in Table 2 represents counts of the frequencies (columns 5–7) of specific SD illusions (column 3) ranked (column 1) by those most experienced across participants (column 4). The frequency classifier “Frequently: >25% of all sorties” had no reports from any participants and is thus not reported here. Reports were consolidated across airframes, as airframe was not a focus of the verification or interview questions. However, because participants were asked to report SD experiences on all airframes they had experience with, there were instances where a single participant reported instances of a specific illusion on multiple airframes. When this was the case, the report of greater frequency was used in the total count seen in Table 2.

**TABLE 2:** Ranking of reported SD events (N = 13). V = visual, B = body-sense, D = display, M = miscellaneous. No reports were made under the “Frequent >25% of all sorties” category.

Rank	Type	Illusion	% participants	N occasional	N seldom	N rarely
1	V	Loss of horizon (atmospheric conditions)	76.9	2	4	4
2	V	Black-hole approach	76.9	0	2	8
3	B	G excess	76.9	1	2	7
4	B	The leans	76.9	0	4	6
5	B	Coriolis illusion	69.2	0	3	6
6	M	Distraction, task saturation or loss of SA	69.2	1	3	5
7	V	Misleading altitude cues from ground texture (e.g., small trees)	61.5	1	2	5
8	V	False horizon (sloping clouds or terrain)	53.8	0	1	6
9	V	Inappropriate use of the sun, moon or northern lights as vertical cue	46.2	0	2	4
10	V	Loss of horizon (brown out, white out, spray out)	46.2	1	1	4
11	D	Misinterpreting information on heads down displays	46.2	0	1	5
12	D	Roll reversal error	46.2	0	0	6
13	M	Giant hand illusion	46.2	0	1	5
14	V	Dip illusion (misjudgment of position in night trail formation)	38.5	0	2	3
15	V	Autokinesis	38.5	0	3	2
16	V	Inability to read instruments after recovery from a flight maneuver	38.5	0	0	5
17	B	Elevator illusion	38.5	0	3	2
18	B	Somatogravic illusion (sense of pitching *up* during acceleration)	38.5	1	2	2
19	D	SD due to (proven) instrument malfunction	38.5	0	0	5
20	M	SD caused by poor crew coordination	38.5	0	0	5
21	B	Graveyard spiral	30.8	1	1	2
22	B	Somatogravic illusion (sense of pitching *down* during decceleration)	30.8	1	1	2
23	V	Vection (yaw sensation caused by anti-collision lights in clouds/fog)	23.1	1	1	1
24	B	Graveyard spin	23.1	0	0	3
25	D	Misinterpreting information on heads up display (HUD)	23.1	0	0	3
26	D	SD while using night vision goggles (NVG)	23.1	0	1	2
27	V	SD while using a drifting/descending aerial flare as a reference	15.4	0	0	2
28	V	Vertigo caused by flickering lights	15.4	0	1	1
29	B	False sense of inversion, e.g., after abrupt level off	15.4	0	0	2
30	B	Undetected drift or descent in hover (RW/VSTOL only)	15.4	1	1	0
31	D	SD using FLIR or other targeting aids	7.7	0	0	1
32	M	Feeling of detachment (high altitude, absent horizon)	7.7	0	0	1
33	D	SD caused by HMD (e.g., JHMCS, ANVIS)	0.0	0	0	0

Of the top 10 most reported SD experiences, six to eight are shared with the most recent surveys conducted by Pennings et al. (2020) and Lewkowicz and Biernacki (2020), including distraction/task saturation, loss of horizon, and black hole approaches. In total, eight illusions were reported by more than 50% of participants (Table 2): four related to visual illusions, three related to body-sense illusions, and one characterized miscellaneous (distraction/task saturation).

Total SD reports by airframe are shown in Table 3. Normalized by the number of participants responding within each airframe, participants experienced an average of 18 Fast-Jet (N = 9), 12 Rotary-Wing (N = 2), 11 Single-Engine (N = 10) and 20 Multi-Engine (N = 4) of the 33 listed SD illusions ranging from one to two episodes only (Rarely) to 5%–25% of all sorties (Occasional) throughout their career (note one illusion, undetected drift/descent during hover, is only applicable to RW).

**TABLE 3:** Total and average number of SD reports per airframe per subject experience.

Airframe	Total SD reports	# Subjects reporting on airframe	Average SD reports per subject
Fast-jet	159	9	18
Rotary wing	23	2	12
Single-engine	112	10	11
Ulti-engine	80	4	20

#### 3.1.2 SD training

All participants received classroom SD training at some point in their career, and 11 of 13 received either ground-based demonstrations, in-flight demonstrations, or both in addition to classroom sessions (with participants A and F only receiving classroom and refresher trainings). On a 7-point Likert scale with anchors of 0 (no value), 4 (satisfactory) and 7 (excellent in all respects), participants rated their SD training to date at an average of 4.2 ± 1.4 standard deviations, with only three participants reporting training as lower than satisfactory. This aligns with recent reports of higher than average satisfaction with SD training (Pennings et al., 2020), and a majority of pilots and navigators rating it from satisfactory to excellent (Lewkowicz and Biernacki, 2020). Taken together with the breadth of experience of the pilots, these results demonstrate a sufficiently representative sample of the populations involved in past SD survey investigations with a comprehensive perspective for the follow-up interview.

### 3.2 Semi structured interview

The thematic analysis of interview data for the thirteen participants produced notable trends within all *a priori* identified gaps in knowledge related to the experience of SD. Analysis of emergent themes centered on specific aspects of SD training that positively impacted the experience and response to SD events in flight (or limitations of SD training), general cognitive and physical responses throughout the duration of SD events, and important considerations for possible SD-specific CMs. The most relevant and accordant reports across participants are presented here. Themes and conclusions drawn from aggregated responses represent generalized trends related to the research gaps, and do not attempt to disambiguate potential effects from nuanced factors such as differences between airframe or fidelity of motion-based simulation environments. Within the subsequent sections, subscripts denote participant IDs Table 1 associated with aggregated responses. Quotes provided within the text and tables were paraphrased for clarity and brevity while retaining the original meaning of the participants’ reports.

#### 3.2.1 Positive transfer of training: Effectiveness

When speaking to how SD training explicitly impacts real SD events experienced in-flight, eleven of thirteen participants reported that training facilitates recognizing SD when it is occurring, and might motion the duration it goes unrecognized by understanding the conditions where it is likely to occur _(B,D,E,F,G,H,I,J,K,L,M)_. This may have indirect effects of shortening recovery from an SD event, as put succinctly by Participant F: “*The quicker you realize something’s up and you’re in trouble, the easier you can get out of it*.”

With respect to how SD training influences SD event response upon recognition, seven of eight participants _(C,D,F,H,J,L,M)_ reported that awareness and anticipation are the pilot’s most effective tools to avoid and overcome SD episodes. Developing a basis of awareness and anticipation of SD events were interpreted to be goals of training _(F,H,G,L,M)_, suggesting effective delivery of the information provided in SD training environments. Along with these reports it was noted that awareness and anticipation are learned and reinforced through:

- Formal SD training (frequency on order of years, including refresher courses)
- Real SD experiences (frequency typically on order of days to months)
- Mission (de)briefings (occurrence dependent on domain/profession)

Nine participants _(C,E,F,G,H,I,J,L,M)_ commented specifically on the transfer of training from simulator- and in-flight SD demonstrations. All nine suggested physical demonstrations (i.e., sensory exposure) are more useful than classroom experience alone. However, five of these participants _(G,H,L,J,M)_ further suggested that coupled training, where simulator- and/or in-flight demonstrations follow literature-based classroom training, is most effective in understanding the experience and implications of SD events. One specific example provided was “*the first time I was setup to have a blackhole approach. We had discussed it but I had never seen it. So when I did see that because of training, I realized I may not be able to trust my visuals even though it was clear”* (L). These reports were more commonly associated with demonstrations of visual illusions, as opposed to somatosensory exposure.

#### 3.2.2 Positive transfer of training: Limitations

There were two key limitations defined from analysis of the interview reports. The first limitation comments on the fidelity (and usefulness) of ground-based simulator and in-flight SD training scenarios. Two participants _(F,L)_ reported that demonstrations of visual-based illusions are better “*since you get realistic visual examples, as compared to simulator somatosensory replication. It’s hard to give a realistic somatosensory example*” (L). Participant H estimated “*10%–15% of the time they recreate [a somatosensory, or body-sense illusion] so when it happens it’s like ‘that’s exactly what I remember’.*” Additionally, Participant I suggested that “*[simulating a somatosensory, or body-sense illusion] is not helpful, and it’s provocative when visual and vestibular stimulation is incongruent. When they’re congruous, disorienting stimuli, however, it is helpful regardless of if it’s operationally realistic*.” These reports suggest that while it may be feasible to teach pilots how to recognize and respond to some specific, commonly experienced visual illusions, somatosensory exposure is only marginally helpful in recognizing specific SD events in real flight. Given the inherent inertial constraints of ground-based flight simulators (Lee, 2017), these reports are not surprising.

Similarly related to comments on simulator cueing fidelity were reports regarding the psychology of training scenarios as compared to real events. Both for simulator- and in-flight demonstrations, participants reported that the psychology related to the expectation _(C,G)_ and consequences _(G,M)_ of SD events in real flight do not exist in training scenarios. Participant C described the presence of expectation as “*true SD which I have experienced in flight is insidious. It sneaks up on you slowly. Even if [the demonstration] goes the way it’s supposed to in the training scenario, you know it’s coming,*” while Participant M noted that “*there was something in the back of my mind that ‘it was a still a simulator’ versus the actual aircraft. I think it may have made the training more impactful if I could have done something wrong and gotten into real trouble.*” Ground-based simulators, and training simulator scenarios are inherently limited with respect to replicating the motion and expectation (or lack thereof) associated with genuine SD events.

#### 3.2.3 Utility of classifying “SD illusions”

Training does seem to impact most pilots’ ability to identify broadly, the underlying causes of the SD event _(C,D,E,F,G,H,J,L,M)_, if not a specific illusion or illusion class (e.g., visual vs. vestibular/somatosensory, acceleration vs. rotation) – e.g., “*When I experienced SD, I would not have been able to put a name to it. It’s more ‘this is the one caused by acceleration and it’s making me feel pitched up and I need to focus on instruments*” (D). However, it appears that in the majority of cases knowing the specific illusion does not substantially aid the pilot in handling and/or overcoming the event – “*you have to have somewhere to go to get through logically rather than having a gut feeling. You will fool yourself*.” (F).

The latter point is not surprising given the reports that SD training typically teaches how to recover from unusual attitudes, as opposed to prescribed responses based on a specific illusion _(A,B,C,D,G,H,J,L,M)_. Through experience, some pilots may develop specific control strategies for specific situations such as “*when you go on a missed approach in weather, don’t push throttle forward so fast. Do it more gradually or [a somatogravic illusion] is going to happen*” (D). There were reports of instances where recognizing a specific illusory phenomenon was beneficial, if not necessary, for recovering from the event:

- Switching hands or transferring control to co-pilot during a Giant Hand illusion _(G,L)_
- Not overcorrecting pitch angle during Somatogravic illusions on high-G takeoffs _(D)_
- Understanding loss of airspeed is due to autorotation (specific to rotary wing) _(B)_

Based on these reports, there may be utility in instructing pilots on prescriptive recovery strategies for specific SD scenarios. However, a general ability to deal with unusual (and inherently stressful) events is applicable across most SD experiences.

#### 3.2.4 Psychological and physiological response to SD

There were unanimous reports that SD training influenced the style of response upon recognition of an existing SD event. When asked to describe a general response upon recognition of SD and whether it is analytical or intuitive, participants all spoke to at least one of three distinct “stages” of psychological and physiological experience.

While a variety of language was provided in the accounts of SD experiences there was accordant meaning of the language used for each stage of the response. The stages described are:

1. A habitual or instinctual reaction to move their attention to instruments _(A,B,C,D,E,F,G,H,J,L,M)_, not react immediately _(G,I,L,M)_, ignore body sensations (or “seat-of-the-pants”) _(A,C,D,E,F,G,I,J,K,L,M)_, and keep their head stationary _(E,G,L,M)_
2. An analytical investigation of the aircraft state _(A,B,C,D,F,G,H,I,J,K,L)_
3. An intuitive or calculated execution of a control response (if warranted) _(B,C,D,F,G,H,I,J,K,L)_

Participants noted that SD training and experience engrain the initial habitual/instinctual reaction to transition attention to the instruments, as opposed to falling victim to additional SD by trusting outside-visual references or bodily perceptions. In all accounts described, the secondary stage involving an analytical investigation of the aircraft state occurs before any control response, e.g., “*Generally [I] stay calm, confirm something is going on there, sensory conflict does exist, and then just recover the aircraft*” (L). Even in the case where participants recognized a substantial discrepancy between actual and perceived aircraft state, a brief analysis of the true state was performed before responding with control input. In these kind of cases, participant G described it as “*If I realized I had a flight parameter significantly different than what I was planning, it’s like ‘knock it off, recover the aircraft, and then figure things out’*”. In this example, “*figure things out*” refers to assessing how they came to be disoriented to begin with, and recovering psychologically and physiologically. Finally, the descriptors “intuitive” and “calculated” participants used to characterize the third stage of the response appear to conflict in meaning without additional context. However, those who described it as “calculated” were referring to executing the appropriate control inputs (or recovery procedure), while those who responded with “intuitive” referred to the impact of training on the ability to execute the necessary control actions. Participant B’s explanation using both descriptors highlights this difference: “*calculated in that you’re trying to think of the proper procedure and what airspeed should be … Intuitive to the point of, it’s not like ‘oh, what do I do with my hands’*”. These reports illustrate a common chronological response to recognizing and responding to SD experiences in flight. The stages of this response were not reported to be taught through training, and may serve as an educational tool to improve the positive transfer of training. They can also be associated with stages in the temporal dynamics of SD events to provide more context on the typical durations and impacts of SD events on pilots.

#### 3.2.5 Temporal dynamics of SD events

Recall Benson’s definition (Benson et al., 1999) of SD as a human’s, “*failure to correctly perceive attitude, position, and motion [of the aircraft]*.” It follows that in most cases, if a pilot recognizes a discrepancy between the actual aircraft state and their perception, there will be some amount of time leading up to recognition of where the pilot’s perception diverged from reality. This is supported by 11/13 participants estimating a duration, or range of durations of Type I (unrecognized) phases of SD events they experienced in flight. It is important to note that unrecognized disorientation, at least with the current understanding, cannot be externally measured, nor is it directly perceived by the pilot (by definition). Therefore, the estimates provided in this investigation are inherently subjective and retrospective. Table 4 lists the quotes from participants related to their estimates of the durations of Type I phases of SD events.

**TABLE 4:** Participant quotes related to estimates of the durations of Type I phases of SD events.

Participant	Supporting quote(s)
A	*“It was less than a minute”*
B	*“…on the order of 10–15 s at most”*
C	*“directly tied to the last time you looked at gyro or ADI. So on the order of seconds, at longest 15 s”*
D	*“almost immediately you realize somethings going on. So you do not follow what you think is happening”*
F	*“…if all that was a minute, maybe 20 s of that”*
G	*“There’s exceptions, but in general it’d be a few seconds”*
H	*“…5 or 10 s max. Most times probably not 10 s”*
I	*“zero to a lot of time”*
J	*“I do not think I’ve ever had a situation where it’s over 30 s”*
L	*“some of those [low frequency, “non-concerned” events] could be upwards of minutes”*
M	*“some within a minute of onset”, “looking back it was probably a few minutes”*

Although there were multiple reports that Type I phases can be on the order of minutes, most participants related the duration to cross-check frequency. With good cross-check habits, most phases of Type I SD are likely to be on the order of 1–15 s. This comes with exceptions that this phase can be longer (e.g., distraction, inconsistent cross-check habits), or essentially non-existent. In some situations participants estimated that when the potential for an SD event was predicted, but could not be avoided, there was effectively no phase of Type I unrecognized SD during the event. Specifically, these reports were with respect to somatogravic (pitch-up) illusions on high-G takeoffs _(H)_, descending into a brown-out scenario _(B)_, and flying at night over water (lights on coastline, strobe reflection off of water) _(B)_.

Participants were also asked to estimate the durations of Type II/III phases of SD events following recognition of existing SD. All reports related directly to one of the three stages aforementioned as the typical psychological and physiological response to recognizing SD. First, six participants made comments relating to possible “periods of ambiguity” following recognition and preceding any additional perception or action. There was unanimous agreement across reports that the recognition of SD and habitual/instinctual reaction to move their attention to the instruments is abrupt, without any period of ambiguity. Table 5 lists the quotes from participants related to their estimates of this first stage of response to the phase of Type II/III SD, and suggests most durations of this stage are on the order of 1’s of seconds.

**TABLE 5:** Participant quotes related to estimates of the durations of the first stage (habitual/instinctual reaction to move attention to instruments) of the psychological and physiological response during a Type II phase of SD.

Participant	Supporting quote(s)
B	*“until you recognize it everything feels normal”*
C	*“There was cognitive dissonance when I got view of the ground … but it seemed like it only took a second or portion of a second to snap back in”*
D	*“almost immediate”*
H	*“almost immediately you realize somethings going on. So you do not follow what you think is happening”*
J	*“You have not recognized you’re disoriented, and when you do that’s when you go ‘wow how long did I not realize we were doing that’, and that’s when it’s kind of abrupt”*
L	*“[In] most instances, there’s no period of slight ambiguity … [it’s] kind of like a step function. Everything’s normal, and then something is weird”*

Ten participants provided reports relating to the durations of the second stage of response to the phase of Type II/III SD involving the analytical investigation of the veridical aircraft state (Table 6). Reports ranged from seconds to upwards of a minute, and suggest this stage of response may typically last on the order of 1’s to 10’s of seconds. From specific accounts of SD experiences, this duration can be influenced by the ability to rapidly interpret instruments (i.e., in the case of highly trained pilots), the perceived severity of consequence of being disoriented in their current state (e.g., flying fast, in close proximity to ground, *etc.*), and the ability to recognize environmental or situational factors that contributed to eliciting the SD event.

**TABLE 6:** Participant quotes related to estimates of the durations of the second stage (analytical investigation of aircraft state) of the psychological and physiological response during a Type II phase of SD.

Participant	Supporting quote(s)
A	*“that one time it was about a minute, other times were just a few seconds”*
C	[Interviewer: so that was seconds to tens of seconds after recognition to deal with that disorientation] *“Yep, correct.”*
D	*“It was like ‘oh I can look at airspeed, I need to pull throttle up and nose up’, that will happen fairly quickly”*
F	*“Usually resolves it within seconds. It may not fix your head”*
G	*“You can start second-guessing like ‘crap I am disoriented’, and ‘okay now I got to figure out which way to go’”*
H	*“Yeah I’d say probably less than 10 s usually for the ones I’ve experienced”*
J	*“No it’d be maybe tens of seconds, like 20 s”*
K	*“on the order of seconds”*
L	*“for most events I’ve seen, it’s in the seconds, two-digit seconds … say 5–30 s”*
M	*“most of the time it’s a fraction of a minute”*

For the third and final stage of the response to the phase of Type II/III SD involving an intuitive/calculated response, only two participants provided reports directly related to the estimated durations (Table 7). They suggest that, if a control response is warranted because the true aircraft state has deviated from the intended state, this response stage typically occurs on the order of 1’s of seconds. While there were no additional reports explicitly commenting on the duration of control responses, accounts of recoveries from specific SD events experienced support this finding. For example, participant D described a somatogravic illusion on a missed approach, and once the analytical investigation was complete, “*[they] went, ‘oh man my airspeed’, and [they] pulled the throttle back and everything was fine*.” Most control corrections described in vivid accounts of SD experiences are plausible to complete on the order of seconds, with the caveat of one account where the pilot’s intended heading had drifted by 90° due to a “Leans illusion”. Correcting heading angle is a function of turn radius, which is influenced by the airframe.

**TABLE 7:** Participant quotes related to estimates of the durations of the third stage (intuitive/calculated control response, if warranted) of the psychological and physiological response during a Type II phase of SD.

Participant	Supporting quote(s)
H	*“If there’s no human in danger … you can slowly and smoothly get yourself out of it. If you try to snap out of it, that can disorient yourself even more”*
L	*“usually in most situations you’re immediately correcting back to that straight and level”*

#### 3.2.6 Psychological and physiological recovery from SD events

The three stages of response to recognizing SD events are in the context of maintaining appropriate control of the aircraft, whether that involves avoiding inappropriate control inputs or taking corrective actions. The psychological and physiological impacts of experiencing SD, however, can have effects on a longer time scale (unanimous, Table 8). These effects include cognitive impacts such as startle and confusion, and physical impacts such as sustained illusory somatosensory perceptions. The duration was noted to be (i) longer with a persisting presence of disorienting stimuli _(C,G,H,M)_ and based on how “strong” (or convincing) the illusion was _(F,L)_, (ii) shorter given an expectation of future motion (i.e., Pilot-in-command vs. Second-in-command) _(L)_ and the opportunity to reduce inertial motion input _(E,H,L)_ (e.g., reduce head movement), and (iii) context-dependent based on whether the SD is due primarily to visual-based or somatosensory-based illusory stimuli _(C,H,L,M)_.

**TABLE 8:** Participant quotes related to the psychological and physiological recovery period following the recognition and experience of SD events.

Participant	Supporting Quote(s)
A	*“the way it felt like when you’re in the midst of doing it, it just felt like it was forever. “am I ever going to get out of this” and after fighting it and fighting it, finally it went away”*
B	*“I want to say correcting [aircraft attitude occurred] prior to the bodily sensation going away”*
C	*“I feel like it could happen up to 15 to 30 min if for an entire portion of the flight my body keeps wanting to turn left and I have to keep correcting right. And each of event of letting it deviate and bring it back may only be a few seconds, but of the event itself, of the dissonance between what your body things a/c is doing and what it’s actually doing can be persistent the whole time you’re IMC”*
D	*“Not suddenly, it gradually went away after about 30 s”*
E	*“Long enough to do the action of stop, focus and it usually cleared up for me”*
F	*“I’ve been through 2 events where it might have been 20–30 s later that my head finally righted itself to match the instruments”*
G	*“Could last a while, and you might have to fly most of the mission on wing before finding clear air”,* Interviewer: [so this could be on the order of minutes, or tens of minutes] “*yeah, especially flying on the wing”*
H	*“maybe 25% of the time, it takes you getting the airplane to a point where you settle down and your gyros get caged and your normal”, “A lot of times a disorientation, like the visual one … As soon as you realize that’s not what you’re looking at … it’s just kind of like okay where is it? Okay it’s over there.”*
I	*“Situation dependent. Something like leans in VFR is a big deal”*
J	*“Oh yeah, definitely, definitely. [The residual feeling of SD] is going to last for a little bit”*
K	*“mostly after you accept it all and forget [your] ears and how things felt”*
L	*“visual illusion, goes away pretty quick. Somatosensory though it could last a minute to 2 minutes. Intensity decreasing”, “might persist depending on how strong the illusion is”, “think it was longer when not active* (pilot-in-command) *because I could not predict future movement of the aircraft”, if you’re able to, if you’re flying straight and level for a minute and a half that helps reset everything”*
M	*“because I had discrepancy between body sense and the instruments. It took maybe a few minutes for me to overcome that”, “I did not get a proper sense of orientation until we broke out on final approach probably 20 min into the event”*

Taken together, these reports suggest psychological and physiological recovery from experiencing SD typically takes on the order of 10’s of seconds to 10’s of minutes after execution of a control response (if warranted). Most reports estimating the duration of recovery in the minutes to 10’s of minutes range are related to the persistence of disorienting stimuli after recovering the aircraft. Specific accounts included flying on the wing in formation in Instrument Flight Rules (IFR) conditions _(G,H)_, flying through or above clouds for extended periods of time _(C,M)_, and flying on a dark moonless night with sparse coastal lights and stars _(C)_. Most of these specific accounts involve flying in IFR, and are succinctly summarized by Participant C’s report that “*the dissonance between what your body thinks the aircraft is doing, and what it’s actually doing can be persistent the whole time you’re in IMC* (Instrument Meteorological Conditions, or IFR).”

The persistence of psychological and physiological effects were not reported as directly related to increased difficulty of performing necessary control input(s). Typically, participants reported that they maintained instrument-flying until perceptual recovery was complete and the disorienting stimuli was no longer present. Language surrounding these reports suggest there is heightened workload during these periods with increased attention for monitoring instrument information, such as participant A’s report that “*the way it felt like when [I was] in the midst of it, it just felt like it was forever. ‘Am I ever going to get out of this,’ and after fighting it and fighting it, it finally went away.*” Additionally, there were a number of reports suggesting distortion of perceived time throughout the perceptual recovery phase causing events to feel longer in the moment than they are actually estimated to be after recovering _(A,B,D,L,J)_. Finally, there were specific reports of SD events involving multiple SD episodes of similar nature when perceptual recovery was drawn out by the persistence of disorienting stimuli _(C, M)_. These reports described recurring “Leans” illusions and SD due to light fixation at night, and suggest periods of extended perceptual recovery can make pilots more susceptible to recurring SD episodes. However, reports also suggest that the increased attention given to the instruments following an initial SD episode typically results in accurate orientation perception for the remainder of the flight.

#### 3.2.7 Countermeasures for mitigating SD

The final portion of the interview gathered opinions from participants regarding the concept of having active countermeasures (CMs) in the cockpit, specifically aimed at assisting the pilot during SD events. There is high degree of inter-personal differences between opinions of pilots on cockpit technologies, monitoring and data collection. The subject pool of this investigation (N = 13) is not large enough to draw conclusions on aspects of personal preference that may explain differences in reports. Therefore, emergent themes where there was broad agreement across participants will be presented here.

While most participants in this investigation predominately had military experience, two with General Aviation (GA) experience commented on key differences in approaches to SD based on airframe that would influence the CM design. Participant M, with only GA experience recounted that they’ve “*flown planes that you couldn’t recover from spin, and knowing that I flew more conservatively*”. Participant G, with GA, military and commercial experience commented that GA SD events have a greater sense of consequence, “*because recovery has to be early, it’s got to be done right, and I don’t have two yellow handles to pull to get out of this.*” These participants made further comments that amenability to a conceptual SD detection and pilot-aiding system would likely be greater within the GA community. This may be related to the greater sense of consequence reported when in GA aircraft as compared to the additional safety features included in advanced airframes existent in commercial and military domains.

When asked for opinions regarding active CM technologies participants made many suggestions on what might be most useful, from small visual warning lights to specific automation modes. Nine participants reported having experience using an autopilot system, and seven of those participants _(B,C,D,E,G,H,L)_ made an unsolicited suggestion that a pilot-actuated “straight-and-level” autonomy mode would be desirable, as opposed to other strategies for shared control. This was related to perceived (lack of) transparency of autopilot (or shared control) systems, and a desire to maintain full authority of aircraft control. A simple and predictable straight-and-level autonomy mode that is manually triggered appears to be considered within the realm of maintaining full authority of the aircraft control. This may stand as an active countermeasure for an initial line of defense when the pilot recognizes an SD event, but the aircraft is not approaching critical limits with respect to flight envelopes or proximity to terrain.

Of the ten participants specifically commenting on signal detection performance (all except A, I, and K), there were unanimous reports that False Alarms (when a CM is triggered during a period where there is not SD or not enough SD to warrant intervention) would be most detrimental to system utility and would dictate the method of intervention. Participants related this to existing caution and warning systems they had experience with in operational aircraft, such as altitude alerts, landing gear warning horns, and stall-approach warnings. Participant D, with the greatest amount of flight hours of all participants, noted that “*[False alarms are] the issue with any kind of warning system that I experienced in aviation. If it goes off too often you start to ignore it, or disable it,*” and further suggested that “*a warning that isn’t really valid is worse than useless*”. This perspective on signal detection performance directly speaks to the utility of SD detection and pilot-aiding systems that would accepted by pilots for integration into the flight deck.

Finally, eleven participants made reports associated with their perceived amenability to additional data collection for improving performance of a conceptual SD detection and pilot-aiding system, and possibly personalizing the system to their flying habits. For the eleven subjects reporting on this aspect (all but I and K), there was concurrence on amenability to additional data collection with caveats of:

- Levels of anonymity and data access _(B,C,F,G,H,J,L,M)_
- Obtrusiveness of possible required hardware _(D,F,G,J)_
- Intent; as related to goal-oriented SD mitigation versus general monitoring _(A,C,D,F,G,H,J)_

Most reports regarding desirable levels of anonymity in the data, as well as the intent of the data collection for being goal-oriented versus general monitoring of pilot behavior were related to possible professional implications (e.g., FAA license, commercial assignment priority, *etc.*), or the general idea of having an entity continuously monitoring their every action (e.g., “big brother”). Reports related to the obtrusiveness of any hardware required for the data collection were strictly related to comfort and the potential for impact on physical aircraft handling. These reports suggest continued research into future SD detection and pilot-aiding systems can proceed without serious concern for pilot acceptability given concerns stem primarily around data security and comfort which may be context-specific in practice.

## 4 Discussion

### 4.1 Chronology of spatial disorientation events

A principal finding of this investigation was the classification of a general chronology of pilot perceptions and actions experienced throughout SD events. Found through our follow-up interview, pilots generally respond to recognition of SD in three stages: (1) a habitual reaction to move attention or refer to instruments that begins, (2) an analytical investigation of aircraft state, (3) followed by a calculated execution of a control response if warranted. In retrospect, it is unsurprising that this mirrors the stages of cognitive processing (perception, cognition and responding) (Isreal et al., 1980; Wickens, 2008), as well as the USAF’s fundamental “recognize, confirm, recover” procedure taught for getting out of upset attitudes. For the first documented time, however, this investigation has characterized the temporal dynamics of these response stages during SD events in terrestrial aviation.

**FIGURE 2:**
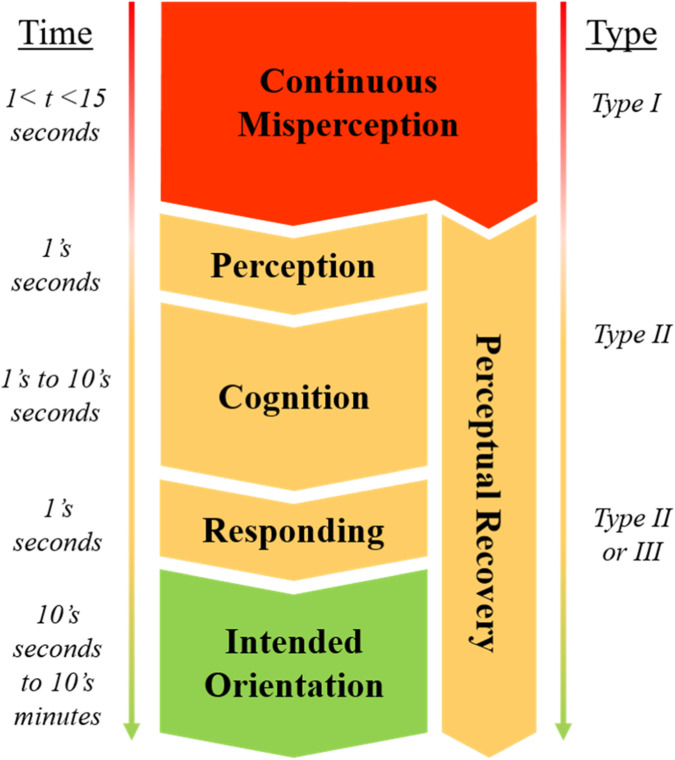
Temporal dynamics of the phases of SD events and stages of response to recognition.

Figure 2 illustrates the entire temporal progression of a typical SD episode. From the moment when a pilot’s perception of aircraft orientation begins to diverge from reality, there is a phase of Type I unrecognized disorientation (red, Figure 2) that generally lasts somewhere between 1 and 15 s with good cross-check habits. The SD episode transitions to a phase of Type II disorientation (yellow, Figure 2) once the pilot recognizes their orientation perception may be inaccurate. At this moment in time, perceptual recovery (right) begins, and the pilot starts performing the three stage response (left) that is directly related to stages of processing:

1. Perception, or the habitual reaction to move attention to instruments upon recognition of potential SD, estimated to be near-instantaneous
2. Cognition, or the analytical investigation of true aircraft state, estimated to range from seconds to tens of seconds
3. Responding, or the calculated execution of a corrective control action if warranted, estimated to be on the order of seconds.

Finally, after a pilot responds to the SD event, and the aircraft is confirmed to be in the intended orientation (green, left, Figure 2), there typically remains a period of perceptual recovery (right). Perceptual recovery refers to the physiological and psychological sensations associated with the SD event. These sensations can continue to produce inaccurate internal estimates of orientation that need to be consciously ignored by the pilot to maintain safe control. Perceptual recovery following the response stage to an SD event typically lasts on the order of tens of seconds, but can persist for tens of minutes, or for the entire duration disorienting stimuli are present. One clear example of this is a “Leans illusion” where there is a sustained bodily sensation of a false gravitational vector. This bodily sensation, even after recognition, can persist for the entire time a pilot is flying with an erroneous or absent ground horizon. Thus, we suggest this still be considered within an SD event, as it may require additional cognitive effort for the pilot to maintain their orientation due to conflicting sensory cues (Angelaki et al., 1999; Clark et al., 2019), potentially putting them at increased risk of another SD episode (Gilbert and Sigman, 2007).

### 4.2 Positive impacts and shortcomings of spatial disorientation training

The initial static survey our participants completed confirmed SD is pervasive across all airframe types, and throughout a pilot’s career. It was observed that illusions of all forms of SD defined as body-sense, visual, display and miscellaneous are commonly experienced across pilots. Although participants estimated the frequency of experiencing classified SD illusions, the interview reports demonstrated that understanding these classes of illusions typically do not influence how SD is perceived and responded to in flight by the pilots themselves. Usually, associating an SD event experienced with a specific illusion type was reported to be retrospective, if at all, and thought about only after the flight/mission was over (e.g., during mission debrief). Therefore, the classification of SD events into unique illusions may only have utility for researchers and instructors, as opposed to the pilot being able to make use of that information *during* an SD event.

While anticipation and awareness of the potential for SD can help a pilot avoid (or greatly shorten) an unrecognized phase of SD (where they cannot avoid experiencing the disorienting stimuli), or avoid the SD event entirely, it does not act as a countermeasure *during* an SD event. Moreover, upset attitude recovery learned through simulator or inflight based training improves a pilot’s ability/skill to make appropriate corrective control actions, but does not influence the perceptual response to recognizing the occurrence of an SD event. These two core focuses of training essentially bookmark the beginning and end of SD events, and do not bridge the gap between recognition and recovery.

Participants explained that knowledge of specific SD illusions learned through classroom-based SD training establishes an awareness of the environments and situations where SD can occur, as opposed to directly aiding the pilot in recognizing and responding to real SD events. Participants reported that specific SD event recovery related to general upset attitude recovery and was not related to recovering from specific SD illusion types. From most accounts of real SD experiences in this study, prescribed responses to recognizing specific SD illusions is not warranted. This argument is motivated by two key factors.

First, there are a number of SD experiences in which the aircraft is in an appropriate orientation, but the pilot’s perception of aircraft state has deviated from their intent (e.g., somatogravic illusion on takeoff). In these situations, a control response upon recognition of SD is not appropriate. Alternatively, there are SD experiences where the aircraft has deviated from the intended orientation, but the pilot’s perception of aircraft state aligns with their intent (e.g., somatogravic illusion in an unintended coordinated turn). In these situations, a control response upon recognition of SD *is* warranted. However, in the complex six degree-of-freedom environment of flight, there is a substantial range of aircraft states (and thus, misperceptions of aircraft state) that can be characterized under a single SD illusion classification.

Therefore, there is also a substantial range of what the appropriate corrective control actions should be for a given SD illusion. Moreover, the appropriate control response is also a function of the phase of flight, mission objectives, environmental constraints, and aircraft capability. For example, a control response to an SD event while flying at low altitude may warrant faster action, a climb in altitude, and constant control input; “*when I’m high up … I’m not as worried, because generally you can pretty much let go of the stick and the aircraft is pretty stable itself. But when you’re flying 500 knots at 200 feet you can’t let go*” (L).

**FIGURE 3:**
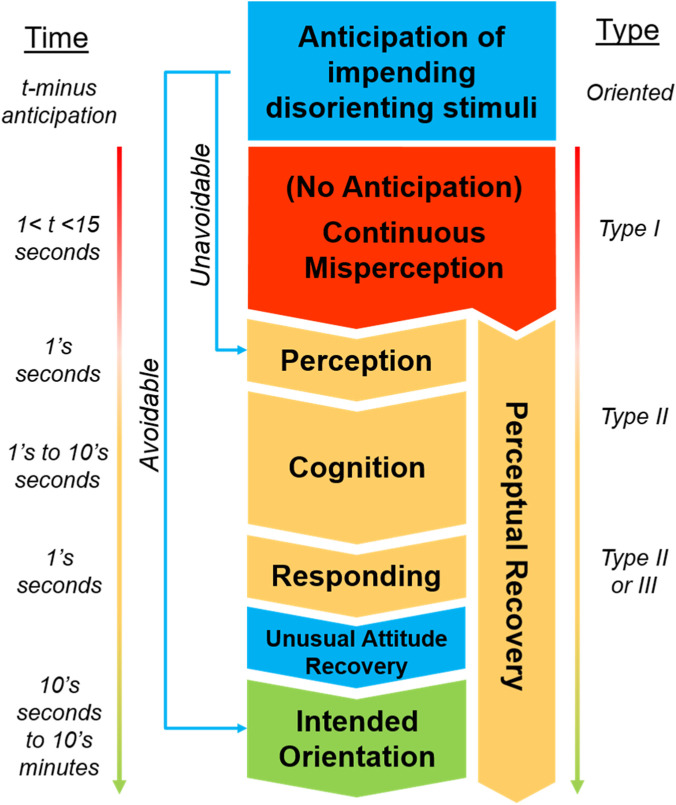
Temporal dynamics of the phases of SD events and stages of response to recognition in the context of how SD training specifically influences the outcomes of the event.

The influence of these training outcomes is graphically depicted in Figure 3, in the context of the temporal dynamics of typical SD events experienced in aviation (Figure 2). Anticipation of the potential onset of disorienting stimuli (top, blue) preceding any potential phase of SD effectively eliminates any duration of unrecognized disorientation (blue arrows bypassing red Type I phase). In the case a pilot cannot avoid the disorienting stimuli due to mission-related factors (e.g., restricted flight plan, flying on wing in formation), they can instantaneously transition to the first stage of response in the Type II recognized phase of disorientation (“Unavoidable” blue arrow). This does not necessarily eliminate the perceptual impact and recovery from the disorienting stimuli, however, but they may be able to remain in the intended orientation while perceptually responding to the event. In the case the pilot has the autonomy to avoid the disorienting stimuli altogether, they can remain in their intended orientation and avoid the event entirely (“Avoidable” blue arrow). Unusual attitude recovery plays a role in the physical execution of the appropriate corrective control action (if warranted) once the pilot determines the appropriate transformation between current and intended aircraft state.

Although the actual response (corrective control action) and perceptual recovery are unique to every SD experience, the standard response and temporal dynamics model shown in Figure 3 offer more insight into strategies for dealing with SD in-flight. Specifically, pilots could be made aware of the potential durations certain stages may take, such that inappropriate or reactionary control inputs are not made. Additionally, strategies to reduce the chance of exacerbating perceptual effects of the SD experience may assist more novice pilots before they develop these strategies themselves in response to experiencing SD.

Finally, with respect to the general effectiveness of SD training, participants who reported receiving simulator and/or in-flight SD training on the survey described the exposure as useful, and most effective following classroom-based SD training. However, it was also reported that it is inherently difficult to genuinely replicate (1) phases of unrecognized SD (Type I), and (2) body-sense/somatosensory SD in training scenarios. The latter limitation can be primarily attributed to intrinsic constraints of ground-based motion simulators.

In summary, we found that SD training is effective in instilling an awareness of the environments and situations where SD is most likely to occur such that pilots could anticipate and/or associate sensory perceptions that result in SD. However, current SD training has little or no impact on the perceptual response to recognizing the occurrence of SD, or any explicit actions taken to recover. Replication of SD illusions through simulator- or in-flight exposure can familiarize pilots with the general feeling of sensory mismatch, but typically does not meaningfully correlate to real SD events experienced in flight except for some specific visual-based illusions. Currently, SD training as a countermeasure assists pilots before SD events occur through anticipation, and after corrective responses are determined following the recognition stages of the event. It does not act as a countermeasure *during* SD events. SD training may be improved by bridging the educational gap between awareness and recovery from unusual attitudes.

### 4.3 Stages of spatial disorientation mitigation

Various concepts for mitigating SD, as detailed in Section 1.2, have been proposed. To date, however, only SD training (a passive, transient CM) has been implemented operationally. The characterization of the temporal dynamics of SD events (Figure 3) is a useful tool for identifying how additional mitigation strategies can most effectively assist the pilot in maintaining safe control of the aircraft. First and foremost, reducing the duration of initial Type I unrecognized phases of SD events has great potential to improve safety, as the vast majority of SD mishaps are a result of the pilot not recognizing, or recognizing the SD event too late to recover ((Stott, 2013; Bles, 2008)). Recall from Section 1.2 that it is unlikely there exists measurable parameters correlated with unrecognized disorientation. However, computational models of human orientation perception have promise for producing estimates of SD being experienced, and critically, do not require the use of unobservable parameters (e.g., control intent). If computational tools capable of estimating pilot SD can be validated, they could be used to transition the pilot to the Type II recognized phase of SD in a shorter duration than if leaving the pilot on their own to recognize their disorientation. Anecdotally, this may have compounding benefits of reducing the duration of the recovery phase since “*the quicker you realize something is up and you’re in trouble, the easier you can get out of it*” (F).

While the perception stage of the recovery process occurs near-instantaneously, the cognitive stage can last for a substantial period of time as the pilot determines the true orientation of the aircraft. An active CM triggered by a model-based estimate of SD, that initially transitions the pilot to the response stage (e.g., auditory alert when SD is detected), can have secondary functions during this time [e.g., SD-recovery displays (Wickens et al., 2007)]. There have been a number of conceptual technologies aimed at delivering effective orientation cues to the pilot such as visual HUDs or HMDs (Previc and Ercoline, 2004; Eriksson, 2009; Wickens et al., 2007), three-dimensional auditory displays (Brill et al., 2016), *etc.* Such active CM concepts have the potential to reduce the duration of the cognitive response stage and reduce the likelihood of inappropriate control inputs. As an alternative to these active CMs being automatically triggered by SD estimates, the results of this investigation suggest that a pilot-actuated “straight-and-level” autonomy mode would be desirable during the cognition stage of recovery and facilitate perceptual recovery. It is worth noting that these CM approaches are not mutually exclusive, and the effectiveness of either is likely influenced by personal preference and situational context.

In most cases, pilots do not need assistance executing an appropriate corrective control action if warranted. In the rarer cases of Type III incapacitated SD, however, there remains potential to prevent resultant mishaps. Two general forms of incapacitation may warrant different approaches:

1. For cognitive incapacitation where a pilot is unable to make appropriate control inputs, but is not physically incapacitated such as during a “Giant Hand illusion”, a pilot-actuated “straight-and-level” autonomy mode would likely be preferred, as opposed to;
2. Automatic transition of control authority to the autopilot due to physical incapacitation of the pilot such as during G-force induced Loss of Consciousness (G-LOC).

With respect to the latter form of physical incapacitation, however, there already exist systems such as the Auto Ground Collision Avoidance System that prevent controlled flight into terrain (Swihart et al., 2011).

Finally, there are no clear recommendations stemming from the temporal dynamics (Figure 3) regarding potential strategies to directly reduce the period of perceptual recovery. However, by shortening the durations of the unrecognized and cognitive recovery phases, it is possible that perceptual recovery will also be faster. To further speculate on potential mechanisms that can shorten the perceptual recovery, there is strong evidence that the Central Nervous System (CNS) performs multisensory integration to produce the most accurate estimates of orientation perception (Angelaki et al., 1999; Clark et al., 2019). This is advantageous in dealing with limitations of physiological sensors, as well as sensory noise. If veridical orientation information is conveyed through active CMs, it is possible that central estimates of orientation parameters would be more accurate and would converge back to reality in a shorter time. The sensory channel through which orientation information is conveyed is likely to influence this potential impact. One could speculate that providing accurate haptic orientation feedback during SD events with somatosensory misperception may assist the pilot in consciously ignoring the false perceptions (McGrath, 2000).

### 4.4 Utilities of mixed methods for investigating spatial disorientation

The findings collected through follow-up interviews with participants completing an initial survey demonstrate the utility of using a mixed methods approach for SD research as compared to periodic isolated surveys. It is worth noting that aircraft investigation also utilizes a mixed methods approach to the analysis of SD events. Accident investigators use all available information to make determinations of underlying causes including quantitative flight data and qualitative voice recordings from black box recovery, and both quantitative and qualitative information related to the pilots’ experience, certifications, general state of health, *etc.* (Newman and FAICD, 2007). In contrast, our mixed methods study focused on investigating experiences of SD from the pilot’s perspective.

Past survey investigations have attempted to collect data relating to the positive impact of training, the experience and response to specific events, and how frequent SD illusions occur between pilot populations (Pennings et al., 2020; Holmes et al., 2003a; Matthews et al., 2002; Lewkowicz and Biernacki, 2020). The limited specificity of the anonymous surveys used in these investigations has resulted in open questions regarding how a pilot’s appreciation of SD training relates to actual experiences, how SD illusions are commonly experienced, and whether SD illusion classifications truly explain the continuum of SD experiences.

We were able to address what aspects of training positively transferred to SD experiences by integrating pilot’s appreciation of training and their reports from the interview (Figure 3). It was found that high appreciation of SD training relates to the awareness and anticipation of SD events that pilots learn through classroom-based lectures and simulator- or in-flight exposure. Although simulator- and in-flight exposure was reported to typically not be representative of similar SD experiences in flight, higher appreciation was also associated with exposure to disorienting stimuli (even if only a Bárány chair demonstration was provided). Lower than average appreciation was associated most closely with reports related to refresher training and/or a lack of simulator-exposure. These reports commented on the repetitive and unchanging nature of refresher courses (typically only involving classroom-based lecture), and the overall awareness gained through real SD experiences as compared to “just talking about it”.

Many participants of this investigation provided reports in the interview on both beneficial and ineffective aspects of SD training, which cannot be captured by a single Likert scale response as included in the WP61 postal survey. We recommend that future investigations involving only a survey consider modifications to the standard WP61 survey that allow participants to rank various aspects of SD training. Future mixed methods investigations could benefit from making similar modifications to the survey in effort to reduce the scope of the interview, and as a result can achieve a larger subject pool for a given amount of effort. A larger subject pool (and targeted recruitment) would enrich the unique results found by using a mixed methods approach, enabling a comparison between pilot populations, airframes, *etc.*, that this preliminary investigation did not attempt to achieve.

With respect to the general chronology of how SD events are perceived and responded to, past surveys have asked for details on the last SD event experienced. This was intentionally not included in our modification of the survey, as it was redundant with structured interview questions. However, results from this study warrant recommendations for future mixed methods investigations. One inherent limitation of the standard WP61 survey is that it asks for details of just one SD experience. This approach cannot capture intra-personal variability and commonality in SD experiences. Moreover, the interpretation of what the open-ended question is asking (e.g., how did you respond to it) does not ensure uniformity in the focus of responses across the subject pool. Results of this investigation suggest there is a common temporal experience for many SD experiences. Future mixed methods investigations may benefit from providing a primer in the survey related to pilot estimates on both typical and specific responses to SD events with quantitative estimates of (and ranges of) durations. Additional context provided through follow-up interviews, can target qualitative correlates to certain aspects of SD such as how underlying causes of the SD event relate to the duration of the Type II and perceptual recovery phases (e.g., common reaction and response when caused by distraction). We suggest that identifying relationships between estimates of typical SD event durations, and other classifiers of SD events is likely to result in more meaningful interpretations of SD experiences from the perspectives of accident investigators and designers of SD detection and pilot-aiding systems.

Finally, the current standard for collecting pilot reports on the frequency of SD events is with respect to classified SD illusions. Our mixed methods approach showed that although pilots are able to classify and group their experiences within the descriptions of SD illusions on the survey (likely because it is the basis of SD training), they rarely perceive them as such in flight. The interview reports showed that even experiences classified under a single illusion can vary substantially, and that identification of a specific illusion rarely influences their perceptual response and recovery. Therefore, we recommend the survey frequency reports not be used in isolation to make specific recommendations on mitigations strategies due to lack of situational context.

## 5 Conclusion

This investigation involved the creation and deployment of a novel mixed methods approach to deepen the understanding of SD experiences of pilots and potential defenses. Confirming previous surveys, we found SD remains pervasive in all aircraft types and throughout a pilot’s career. The SD training pilots receive is appreciated and perceived to be helpful, particularly in the form of better awareness/anticipation which may reduced the duration SD events remain unrecognized.

Upon recognition of an SD event, we found pilots perform a three-stage recovery process involving a habitual reaction to move attention to aircraft instruments, an analytical investigation of aircraft state, and a calculated execution of corrective control actions if appropriate. In a temporal dynamics model, we captured estimated ranges of duration for each phase, suggesting most SD events occur on the order of tens of seconds to minutes, whiel perceptual recovery may persist up to tens of minutes following recovery of the aircraft’s orientation.

The temporal dynamics model presented in this paper provides a basis for informing and optimizing SD countermeasure systems. Specifically, computational SD detection methods have promise in shortening the duration that SD goes unrecognized, which is critical since most accidents caused by SD are resultant of times where the pilot did not recognize the event. Orientation information provided through active countermeasures may be able to minimize the duration of the cognitive stage of recovery. This would also likely result in improved flight safety and may reduce the duration required for perceptual recovery.

Pilots, however, reported that the most important design criteria for creating *useful* active CMs triggered by an SD detection model is the absence (or minimization) of false alarms. This is supported by findings in literature on the impacts of false alarms in aviation and automation systems (Bliss et al., 1999; Dixon et al., 2007). Specifically, it was found false alarm-prone systems hurt overall task performance worse than miss-prone systems, and that false alarms affect both operator compliance and reliance. Additionally, given that SD events are relatively rare with respect to incident rates per number of flight hours, it is logical to assume that occasional misses would be understood and accepted, as compared to a substantial number of false alarms. Results of our interview investigation align with these ideas. Overall, the findings from this investigation directly support the development of computational detection strategies, provide recommendations for CM design and implementation, and highlight areas of SD training that may be able to be improved.

## Data Availability

The datasets presented in this article are not readily available because they include specific and personal accounts provided in interviews that could be potentially identifiable, violating the terms of the IRB approval. Requests to access the datasets should be directed to the corresponding authors.

## References

[B1] Albery W. B. (2007). Multisensory cueing for enhancing orientation information during flight. Aviat. Space, Environ. Med. 78 (5), B186–B190. Available online at: https://pubmed.ncbi.nlm.nih.gov/17547319/. 17547319

[B2] Angelaki D. E. McHenry M. Q. Dickman J. D. Newlands S. D. Hess B. J. (1999). Computation of inertial motion: neural strategies to resolve ambiguous otolith information. J. Neurosci. 19 (1), 316–327. 10.1523/JNEUROSCI.19-01-00316.1999 9870961 PMC6782388

[B3] Benson A. J. (1999). Spatial disorientation - general aspects. Editors Medicine A. Ernsting J. Nicholson A. N. Rainford D. J. (London: Butterworths), 277–296.

[B4] Bles W. (2008). Spatial disorientation training: demonstration and avoidance. (France: North Atlantic Treaty Organization, Research and Technology Organization: Neuilly-sur-Seine Cedex).

[B5] Bliss J. P. Freeland M. J. Millard J. C. (1999). “Alarm related incidents in aviation: a survey of the aviation safety reporting system database,” in Proceedings of the human factors and ergonomics society annual meeting (Los Angeles, CA: SAGE Publications Sage CA).

[B6] Boril J. Smrz V. Petru A. Blasch E. Leuchter J. Frantis J. (2018). “Survey of spatial disorientation and sensory illusion among air force pilots,” in 2018 IEEE/AIAA 37th digital avionics systems conference (DASC), (London, United Kingdom: IEEE).

[B7] Braithwaite C. M. G. (2002) “Spatial disorientation: towards international standardization,” in Spatial disorientation in military vehicles: causes, consequences and cures. Available online at: https://pubmed.ncbi.nlm.nih.gov/9819157/.

[B8] Braithwaite M. G. Durnford S. J. Crowley J. S. Rosado N. R. Albano J. P. (1998). Spatial disorientation in US Army rotary-wing operations. Aviat. Space, Environmental Medicine 69, (11), 1031–1037. 9819157

[B9] Braun V. Clarke V. (2006). Using thematic analysis in psychology. Qual. Research Psychology 3 (2), 77–101. 10.1191/1478088706qp063oa

[B10] Brill J. C. Lawson B. Rupert A. H. (2016). “Countermeasures for loss of situation awareness: error filtering strategies for 3-dimensional audio,” in 2016 IEEE aerospace conference, (IEEE).

[B11] Cheung B. (2013). Spatial disorientation: more than just illusion. Aviat. Space, Environ. Med. 84 (11), 1211–1214. 10.3357/asem.3657.2013 24279238

[B12] Cheung B. Money K. Wright H. Bateman W. (1995). Spatial disorientation-implicated accidents in Canadian forces, 1982-92. Aviat. Space Environmental Medicine 66 (6), 579–585. Available online at: https://pubmed.ncbi.nlm.nih.gov/7646410/. 7646410

[B13] Clark T. K. Newman M. C. Karmali F. Oman C. M. Merfeld D. M. (2019). Mathematical models for dynamic, multisensory spatial orientation perception. Prog. Brain Research 248, 65–90. 10.1016/bs.pbr.2019.04.014 31239146

[B14] Collins D. L. Harrison G. (1995). Spatial disorientation episodes among F-15C pilots during operation desert storm. J. Vestibular Research Equilibrium Orientation 5 (6), 405–410. Available online at: https://pubmed.ncbi.nlm.nih.gov/8589852/. 8589852

[B15] Creswell J. W. (2014). A concise introduction to mixed methods research. California: SAGE Publications, Inc.

[B16] Creswell J. W. Creswell J. D. (2018). Research design: qualitative, quantitative, and mixed methods approaches. Fifth. (Thousand Oaks, California: SAGE Publications, Inc.).

[B17] Dixon S. R. Wickens C. D. McCarley J. S. (2007). On the independence of compliance and reliance: are automation false alarms worse than misses? Hum. Factors 49 (4), 564–572. 10.1518/001872007X215656 17702209

[B18] Dixon J. B. Endsley T. Clark T. K. (2019). “A mathematical model-based metric of spatial disorientation for use in triggering active countermeasures,” in Proceedings of the human factors and ergonomics society annual meeting (Los Angeles, CA: SAGE Publications Sage CA).

[B19] Durnford S. J. DeRoche S. L. Harper J. P. Trudeau L. A. (1996). Spatial disorientation: a survey of US Army rotary-wing aircrew. (Fort Rucker, AL: Army Aeromedical Research Lab). Available online at: https://apps.dtic.mil/sti/tr/pdf/ADA307857.pdf.

[B20] Edgington E. S. (1967). Review of the discovery of grounded theory: strategies for qualitative research. Can. Psychologist/Psychologie Canadienne 8a (4), 360. 10.1037/h0083159

[B21] Eriksson L. (2009). Toward a visual flow integrated display format to combat pilot spatial disorientation. Int. J. Aviat. Psychol. 20 (1), 1–24. 10.1080/10508410903415922

[B22] Gaydos S. J. Harrigan M. J. Bushby A. J. (2012). Ten years of spatial disorientation in U.S. Army rotary-wing operations. Aviat. Space Environ. Med. 83 (8), 739–745. 10.3357/asem.3196.2012 22872986

[B23] Gibb R. Ercoline B. Scharff L. (2011). Spatial disorientation: decades of pilot fatalities. Aviat. Space, Environ. Med. 82 (7), 717–724. 10.3357/asem.3048.2011 21748911

[B24] Gilbert C. D. Sigman M. (2007). Brain states: top-down influences in sensory processing. Neuron 54 (5), 677–696. 10.1016/j.neuron.2007.05.019 17553419

[B26] Groen E. L. Bos J. Houben M. (2016). “Pilot perception model supports the analysis of vestibular illusions in flight accidents,” in AIAA guidance, navigation, and control conference.

[B27] Holmes S. R. Bunting A. Brown D. L. Hiatt K. L. Braithwaite M. G. Harrigan M. J. (2003a). Survey of spatial disorientation in military pilots and navigators. Aviat. Space Environ. Medicine 74 (9), 957–965. Available online at: https://pubmed.ncbi.nlm.nih.gov/14503674/. 14503674

[B28] Holmes S. R. Bunting A. Bostock S. Brown L. Hiatt K. Braithwaite M. (2003b). Preliminary survey of spatial disorientation in UK military pilots and navigators. (Farnborough, United Kingdom: Qinetiq Ltd Centre For Human Sciences).

[B29] Isreal J. Chesney G. L. Wickens C. D. Donchin E. (1980). P300 and tracking difficulty: evidence for a multiple capacity view of attention. Psychophysiology 17 (3), 259–273. 10.1111/j.1469-8986.1980.tb00146.x 7384376

[B30] Johnson R. B. Onwuegbuzie A. J. Turner L. A. (2016). Toward a definition of mixed methods research. J. Mixed Methods Res. 1 (2), 112–133. 10.1177/1558689806298224

[B31] Kuipers A. Kappers A. van Holten C. R. van Bergen J. H. W. Oosterveld W. J. (1990). Spatial disorientation incidents in the RNLAF F 16 and F 5 aircraft and suggestions for prevention. AGARD Situational Aware. Aerosp. Operations (SEE N 90-28972 23-53), 25–40. Available online at: https://apps.dtic.mil/sti/tr/pdf/ADA223939.pdf.

[B32] Kynor D. B. (2002). Disorientation analysis and prediction system. (USAF: WPAFB, OH), p. 77.

[B33] Lee A. T. (2017). Flight simulation: virtual environments in aviation. (Oxfordshire, United Kingdom: Routledge).

[B34] Lewkowicz R. Biernacki M. P. (2020). A survey of spatial disorientation incidence in Polish military pilots. Int. J. Occup. Med. Environ. Health 33 (6), 791–810. 10.13075/ijomeh.1896.01621 33029026

[B35] Malcolm R. (1984). The Malcolm horizon: history and future. NASA. Dryden Flight Research Center Peripheral Vision Horizon Display (PVHD), 11–40.

[B36] Matthews R. S. Previc F. Bunting A. (2002). “USAF spatial disorientation survey,” in Paper to be presented at the spatial disorientation in military vehicles: causes, consequences and cures, a symposium organised by the NATO human factors and medicine panel (Spain: La Caoruna).

[B37] McGrath B. J. (2000) “Tactile instrument for aviation,” in Naval aerospace medical research lab pensacola fl.

[B38] Navathe P. D. Singh B. (1994). Prevalence of spatial disorientation in Indian Air Force aircrew. Aviat. Space Environ. Medicine 65, 1082–1085. Available online at: https://pubmed.ncbi.nlm.nih.gov/7872907/. 7872907

[B39] Newman D. G. Faicd A. (2007). An overview of spatial disorientation as a factor in aviation accidents and incidents. Aust. Transp. Saf. Bur. Canberra City. Available online at: http://www.dviaviation.com/files/45147727.pdf.

[B40] Newman M. Lawson B. D. McGrath B. J. Rupert A. H. (2014). “Perceptual modeling as a tool to prevent aircraft upset associated with spatial disorientation,” in AIAA guidance, navigation, and control conference.

[B41] O’Neil P. Hamilton B. A. Miller M. (2017). “USAF spatial disorientation prevention: a meta-analytical human systems integration perspective,” in 19th international symposium on aviation psychology.

[B42] Onur C. Bozan A. Pritchett A. (2014). “Computational modeling to predict pilot’s expectation of the aircraft state given vestibular and visual cues,” in 2014 systems and information engineering design symposium (SIEDS) (IEEE).

[B43] Osei-Amanfi M. (2018). A case study exploration of strategies to avoid cloud computing data breaches. (Grand Canyon University; ProQuest Dissertations & Theses Global).

[B44] Paillard A. C. Quarck G. Denise P. (2014). Sensorial countermeasures for vestibular spatial disorientation. Aviat. Space Environ. Med. 85 (5), 563–567. 10.3357/asem.3735.2014 24834571

[B45] Pennings H. J. M. Oprins E. A. P. B. Wittenberg H. Houben M. M. J. Groen E. L. (2020). Spatial disorientation survey among military pilots. Aerosp. Med. Hum. Perform. 91 (1), 4–10. 10.3357/AMHP.5446.2020 31852567

[B46] Poisson R. J. Miller M. E. (2014). Spatial disorientation mishap trends in the U.S. Air Force 1993–2013. Aviat. Space Environ. Med. 85 (9), 919–924. 10.3357/ASEM.3971.2014 25197890

[B47] Previc F. H. Ercoline W. R. (2004) “Spatial disorientation in aviation,” in Progress in astronautics and aeronautics, American Institute of Aeronautics and Astronautics, 203. 576.

[B48] Previc F. H. (2012). The effects of a novel head-mounted symbology on spatial disorientation and flight performance in US Air Force Pilots. (Wright-Patterson AFB, Ohio: School of Aerospace Medicine).

[B49] Raj A. K. Braithwaite G. (1999). The tactile situation awareness system in rotary wing aircraft: flight test results. Curr. Aeromed. Issues Rotary Wing Operations 5.

[B50] Roscoe S. N. (1997). Gateway. (Wright-Patterson AFB, Ohio: Crew System Ergonomics Information Analysis Center), vol 7.

[B51] Rupert A. H. (2000). An instrumentation solution for reducing spatial disorientation mishaps. IEEE Eng. Med. Biol. Mag. 19 (2), 71–80. 10.1109/51.827409 10738664

[B52] Sipes W. E. Lessard C. S. (2000). A spatial disorientation survey of experienced instructor pilots. IEEE Eng. Med. Biol. Mag. 19 (2), 35–42. 10.1109/51.827403 10738658

[B53] Small R. L. Keller J. W. Wickens C. D. Socash C. Ronan A. M. Fisher A. M. (2006). Multisensory integration for pilot spatial orientation. (Boulder, Colorado: Micro Analysis And Design).

[B54] Small R. L. Keller J. W. Wickens C. Oman C. M. Jones T. D. (2011). Modeling and mitigating spatial disorientation in low G environments. (Cambridge, MA: Alion Science and Technology Corp. and Massachusetts Institute of Technology).

[B55] Stott J. R. R. (2013). Orientation and disorientation in aviation. Extreme Physiology Med. 2 (2), 2. 10.1186/2046-7648-2-2 23849216 PMC3710190

[B56] Swihart D. E. Barfield A. F. Griffin E. M. Lehmann R. C. Whitcomb S. C. Flynn B. (2011). Automatic ground collision avoidance system design, integration, & flight test. IEEE Aerosp. Electron. Syst. Mag. 26 (5), 4–11. 10.1109/MAES.2011.5871385

[B57] Tormes F. R. Guedry F. E. Jr (1974). Disorientation phenomena in naval helicopter pilots. (Pensacola, FL: Naval Aerospace Medical Research Lab). 1147873

[B58] Tornero Aguilera J. F. Gil-Cabrera J. Clemente-Suarez V. J. (2020). Determining the psychophysiological responses of military aircrew when exposed to acute disorientation stimuli. BMJ Mil. Health 168, 112–116. 10.1136/bmjmilitary-2020-001417 32205329

[B59] Van Erp J. B. Groen E. L. Bos J. E. van Veen H. A. H. C. (2006). A tactile cockpit instrument supports the control of self-motion during spatial disorientation. Hum. Factors 48 (2), 219–228. 10.1518/001872006777724435 16884044

[B60] Wickens C. D. (2002). Situation awareness and workload in aviation. Curr. Directions Psychological Science 11 (4), 128–133. 10.1111/1467-8721.00184

[B61] Wickens C. D. (2008). Multiple resources and mental workload. Hum. Factors 50 (3), 449–455. 10.1518/001872008X288394 18689052

[B62] Wickens C. D. Small R. L. Fisher A. M. Keller J. W. Socash C. M. (2007). “On line and off-line tools for preventing and analyzing vestibular spatial disorientation mishaps: a summary of the Alion-MA&D/Air force research program,” in 2007 international symposium on aviation psychology.

[B63] Young L. (2003). “Spatial orientation,” in Principles and practice of aviation psychology. Editor Tsang P.S. Vidulich M.A. (Mahwah, NJ: Lawrence Erlbaum).

