# Supplementary material for: Findings from interviews with pilots on spatial disorientation: training, temporal dynamics and countermeasures
Source: Front Physiol. 2026 Feb 10;16:1620737. doi: 10.3389/fphys.2025.1620737 (PMC12929113; doi:10.3389/fphys.2025.1620737)

APPENDIX A: Spatial Disorientation Survey

The purpose of this survey is to gather information from **aircrew** on the types and frequencies of spatial disorientation (SD) experienced during operations. The survey and interview are being conducted in order to guide future research efforts and ultimately **counteract the effects of SD** in pilots and other navigators**.**  The definition of SD for the purposes of the survey and interview is as follows:

**An incorrect perception of your attitude, altitude, position or motion, relative to the Earth’s surface or another aircraft, SUFFICIENT TO AFFECT YOUR PERFORMANCE, SITUATIONAL AWARENESS OR WORKLOAD – HOWEVER SLIGHT THAT EFFECT MAY BE.**

| **Demographic Information** | | | |
| --- | --- | --- | --- |
| Name | Age (Years) | Sex | Current Position and/or Aircraft Flown |
|  |  |  |  |
| Certifications, Ratings & Professional Aviation Degrees (TPS, aerobatic school, etc.) – *List all* | | | |
|  | | | |

*Approximations are sufficient.*

| **Flight Experience** | | | | | | | |
| --- | --- | --- | --- | --- | --- | --- | --- |
| Aircraft Type | Total Hours | Last Year Flown | Pilot-In-Command Hours | Second-In-C Hours | IFR Hours | Combat Hours (*military only*) |  |
| Fast Jet / High Performance (FJ) |  |  |  |  |  |  |  |
| Rotary Wing (RW) |  |  |  |  |  |  |  |
| Single-Engine (SE) |  |  |  |  |  |  |  |
| Multi-Engine (ME) |  |  |  |  |  |  |  |
| Other (spacecraft, glider, lighter-than-air, RPA, etc.) – *List all* |  |  |  |  |  |  |  |

What type of training have you received on SD? None / Lecture / Ground-Demonstration / In-Flight

(*Circle all that apply*)

How long ago was your last SD refresher course (including CRM training)? ________ Years ________Months

Who conducted your last SD training (pilot, physiologist, etc.)?

How do you rate your overall SD training to date?

*Rating Scale (1-7): 1 (no value) to 4 (satisfactory) to 7 (excellent in all respects)*

On the next page, please record how frequently you have experienced each of the listed SD illusions (rows) **for all of the airframes with which you have experience**. Frequency and airframe classifiers are provided at the top of the next page. **Please read each description carefully**. Do not include scheduled in-flight SD demonstrations.

**FREQUENCY Classifiers (*mark airframes as applicable*)**

**N**ever = Never or N/A

**R**arely = 1-2 episodes only

**S**eldom = <5% of **ALL** sorties

**O**ccasional = 5-25% of **ALL** sorties

| **FREQUENCY per AIRFRAME** | | | | | **DESCRIPTION** | |
| --- | --- | --- | --- | --- | --- | --- |
| **FJ** | **RW** | **ME** | **TR** | **Oth** |  |  |
|  |  |  |  |  | Inappropriate use of the sun, moon or northern lights as vertical cue | V  I  S  U  A  L |
|  |  |  |  |  | Sloping horizon – orientation against sloping clouds or terrain |  |
|  |  |  |  |  | Loss of horizon - SD caused by atmospheric conditions blending earth and sky |  |
|  |  |  |  |  | Loss of horizon - SD caused by blowing sand, dust or snow (brown-out/white-out) |  |
|  |  |  |  |  | SD while using a drifting/descending aerial flare as a reference |  |
|  |  |  |  |  | Misjudgment of position or motion in formation trail (e.g. dip illusion) |  |
|  |  |  |  |  | Night approach to a runway with misleading visual cues – black-hole approach |  |
|  |  |  |  |  | Misleading altitude cues from ground texture (e.g., over flat water, small trees, etc.) |  |
|  |  |  |  |  | Apparent motion of a fixed point light source (autokinesis) |  |
|  |  |  |  |  | False sensation of yaw caused by anti-collision light reflecting off cloud/fog |  |
|  |  |  |  |  | Vertigo caused by flickering light – strobe light or sunlight through rotor disc/prop. |  |
|  |  |  |  |  | Inability to read instruments clearly following recovery from a flight maneuver |  |
|  |  |  |  |  | Sensation of rolling/pitching after abrupt head movement in a turn (Coriolis) | B  O  D  Y  S  E  N  S  E |
|  |  |  |  |  | False or exaggerated sense of bank in a high-G turn (G-excess) |  |
|  |  |  |  |  | False sense of upward/downward motion as if in an elevator |  |
|  |  |  |  |  | False sense of inversion – e.g., after abrupt level off |  |
|  |  |  |  |  | Recover from a spin, spin perceived in opposite direction, spin re-entered (graveyard spin) |  |
|  |  |  |  |  | Roll level from coordinated turn, sense roll in opposite direction, re-enter turn (graveyard spiral) |  |
|  |  |  |  |  | Leaning in response to a false sensation of bank after recovery to wings level (the “leans”) |  |
|  |  |  |  |  | False sense of pitching up on take-off or when accelerating in flight |  |
|  |  |  |  |  | False sense of pitching down with abrupt deceleration in flight |  |
|  |  |  |  |  | Undetected drift/descent in the hover **(Rotary/VSTOL ac only)** |  |
|  |  |  |  |  | Perceived inability to make effective input to correct bank angle (like a giant hand holding the wing) | M  I  S  C |
|  |  |  |  |  | Feeling of detachment/no longer being in control of own aircraft (high altitude/absent horizon) |  |
|  |  |  |  |  | SD caused by distraction, task saturation and/or loss of situational awareness |  |
|  |  |  |  |  | Poor crew coordination leading to SD |  |
|  |  |  |  |  | Problems interpreting spatial orientation information on the head-down displays | D  I  S  P  L  A  Y |
|  |  |  |  |  | Problems interpreting spatial orientation information on a head-up display (HUD) |  |
|  |  |  |  |  | Problems interpreting spatial orientation information on a helmet-mounted display (HMD) |  |
|  |  |  |  |  | Erroneous bank correction using any attitude indicator (e.g. roll-reversal error) |  |
|  |  |  |  |  | Disorientation while using night vision goggles (NVG) |  |
|  |  |  |  |  | Disorientation while using forward looking infra-red (FLIR) or other targeting aids |  |
|  |  |  |  |  | Disorientation due to instrument malfunction (proven malfunction only) |  |
| **-** | **-** | **-** |  | **-** | Other disorienting illusions/factors (*please describe*) | O  T  H E  R |
|  |  |  |  |  |  |  |
|  |  |  |  |  |  |  |
|  |  |  |  |  |  |  |

**F**requently = >25% of **ALL** sorties

**AIRFRAME Classifiers**

**FJ** = Fast Jet / High Performance

**RW** = Rotary Wing,

**SE** = Single-Engine,

**ME** = Multi-Engine

**Oth** = Other (spacecraft, glider, etc.)

APPENDIX B: Spatial Disorientation Subject Matter Expert Interview

For the purposes of the survey and this interview, the definition of spatial disorientation (SD) is as follows:

**An incorrect perception of your attitude, altitude, position or motion, relative to the Earth’s surface or another aircraft, SUFFICIENT TO AFFECT YOUR PERFORMANCE, SITUATIONAL AWARENESS OR WORKLOAD – HOWEVER SLIGHT THAT EFFECT MAY BE.**

1. Classification of SD & SD Training

*To assess background and familiarity with SD, whether their perception of SD & types of perceptions are influenced by training, and whether there exists SD episodes, regardless of severity, that do not fall under common classifications/illusions.*

1. Can you provide some more details on the extent of your SD training(s), both initial and refresher courses? (e.g. high-level overview of the topics covered, demonstrations given, fidelity of simulator or in-flight demonstrations, airframes covered, etc.)
2. How do you feel your SD training(s) impacted your ability to:
   1. Recognize the presence of SD?
   2. Identify the type of SD being experienced?
   3. Recover from the SD event?
3. Were there specific aspects of the training(s) that was/were better than others?
4. Do all of your SD experiences fall under the common classifications taught in training and seen on the list in the survey you filled out beforehand? If not, what are the characteristics of these specific events that do not necessarily fit within the common classification?(Common) Dynamics of SD Events

*To better understand how SD progresses after ‘onset’, how the severity & ability to recover relate to the SD progression, and how much of the event is directly perceived & indirectly deduced/estimated by the pilot.*

1. Please think about your most vivid or recent SD event when answering the following questions:
   1. What were your thought processes and actions taken throughout that event?
   2. What cues or events led you to recognize that you were disoriented?
   3. Can you describe the moments preceding the recognition of SD?
   4. How did you ‘get out’ of the feeling of disorientation, or regain an appropriate sense of orientation?
      1. Did you regain an appropriate sense of orientation before or after you corrected the aircraft state?
   5. Approximately how long did it take you from the point of recognition to regain an appropriate sense of orientation (not including additional time needed to correct the aircraft state)? (i.e. duration of the Type II phase of the SD event)
   6. Did you attempt to estimate how long you may have been disoriented, or misperceived the aircraft state, before recognizing it? (i.e. duration of the Type I phase of the SD event.)
      1. [If so] Approximately how long did you estimate you were disoriented without realizing it – from the ‘onset’ of SD to recognition? And at what point during or after the mission did you estimate this? Did this thought process affect how you perceived or dealt with SD following this event?
      2. [If not] Was there a specific reason that you did not think about your perceptions leading up to you recognizing disorientation, or did that simply not cross your mind at the time?
2. Please think about all of your SD experiences when answering the following questions:
   1. When you’re experiencing SD, do you feel overwhelmed? Physically? Mentally? How often, or to what extent?
   2. Do you feel you could provide a general chronology of perceptions, thoughts and actions taken during any SD event?
      1. [If so] Can you detail this typical timeline of events?
      2. [If not] Why do you feel there is not an acceptable description of the chronology of SD events, broadly?
   3. What cues or events, other than those you listed for the specific event, have led you to recognize that you were disoriented?
   4. Other than the action(s)/method(s) you listed for the specific event, what are some other ways you’ve ‘gotten out’ of the feeling of disorientation, or regained an appropriate sense of orientation?
      1. Are there more common actions taken in most scenarios, or is it highly dependent on the event?
      2. Do you feel your actions taken were more ‘intuitive’ or more ‘calculated/analytical’?
      3. Do you feel your SD training had a significant impact on this behavior?
      4. What is the proportion of times you’ve regained an appropriate sense of orientation before correcting the aircraft state to the times you’ve regained orientation only after correcting the aircraft state?
   5. Can you estimate the range of durations (e.g. anywhere from 2 sec to 2 min) that it has taken you from the point of recognition that you were disoriented, to regaining an appropriate sense of orientation (not including additional time needed to correct aircraft state)? (i.e. range of duration of Type II phases)
   6. Can you estimate the range of durations that you’ve judged you were disoriented, or misperceiving the aircraft state, preceding recognition? (i.e. range of durations of Type I, unrecognized phases) Do you feel that the duration of any of the spatial disorientation phases has an effect on your ability to remember or report details of the event, both with respect to the possible unrecognized phase (Type I), and the recognized phase (Type II/III)?
   7. Are there events that have occurred in which you believe you’ve realized your disorientation at the instant of ‘onset’, or does recognition always take being disoriented for some amount of time? In other words, has there ever been a time you felt aware of the SD event from the moment you became disoriented?
3. Do you think you have ever predicted an impending SD event, and avoided experiencing SD by taking corrective action?
   1. If so, please describe the event and list what cues led you to the prediction that you were about to become disoriented.
   2. What corrective actions did you take?
4. Have you ever predicted an impending SD event, but been unable to avoid experiencing SD?
   1. If so, please describe the event and list what cues led you to the prediction that you were about to become disoriented.
   2. How did you attempt to avoid the SD event?
   3. How do you feel predicting the SD event affected your perceptions and actions after becoming disoriented?
5. What aspects of a spatial disorientation event do you use or think about while making a judgement as to the severity of the event?
   1. Do you make estimations on your ‘ability to recover’ from a spatial disorientation event? If so, how and when?
      1. [For Interviewer: e.g., subjective perception of flight safety, , ability to recover, difference in illusory orientation and actual orientation following recognition, estimated time to impact etc.]
   2. [For the aspects listed] If you were to estimate or even hypothesize how [the aspects you’ve listed] might change over time during phases of unrecognized disorientation (Type I)? For example, if you felt there was no risk to flight safety prior to the onset of unrecognized SD, and after recognition you felt there was significant risk to flight safety, how would you estimate that risk progresses from none to significant through the duration of the unrecognized phase? Feel free to use empty space on this interview to depict your thoughts.
      1. Would you say this behavior is generalizable across many of your SD experiences, or is this only representative of a specific incidence of which you were thinking?
6. Views & Opinions on “Tools” and Countermeasure Techniques

*To get a sense of whether there may exist tools or countermeasures that may be robust in the sense of being applicable/helpful in most or all SD scenarios (not only for the purpose of our experimentation, but for recommendations for future pilot aiding systems), or whether the appropriate modality and/or strategy (and thus, countermeasure) that can be helpful varies significantly.*

- 1. What “tools” or strategies do you most commonly employ to fight SD and reorient yourself?
  2. Do you feel the most appropriate or useful tool(s) and/or strategies vary as significantly as the type of SD being experienced?
  3. Could you imagine a tool and/or strategy that may be robust in the sense that it would be at least helpful, but not necessarily optimal, in most or all SD scenarios?
  4. In all of your SD experiences, was there a common bodily sense/modality (e.g. visual, vestibular, auditory, somatosensory, etc.) that you felt was particularly incapacitated or overloaded? One that felt particularly unaffected?
  5. Please imagine that there is a computer system integrated into the flight deck that tracks the motions of the aircraft, yoke and your head/helmet. This system makes predictions of whether or not you are experiencing SD.
     1. First, imagine this system was always reliable when predicting SD; it does not necessarily catch every single SD experience, but every time it predicts SD it is correct.
        1. How amenable would you be to the aircraft taking partial or full control when this system predicted SD? Are there specific tasks that you would always want to maintain complete authority over, or that you would always want the system to take over?
     2. Now imagine this system never misses predicting an SD event; there will never be an actual SD event it does not predict, but there will also be a significant number of predictions when you are not experiencing SD (i.e. false alarms).
        1. How amenable would you be to the aircraft manipulating instruments or displays (e.g. HMD/HUD) based on these predictions in order to draw your attention to your orientation?
        2. If this system was implemented in an aircraft you were flying, what would be an acceptable level of obtrusiveness of a countermeasure like a HUD, that is not taking authority over tasks, but keeping in mind there is a nontrivial number of false alarms? Please use whatever wording you’d like to best describe your thoughts.
  6. For the purpose of developing such a system, would you be amenable to having additional data collected from you while flying, such as head movements, gaze location and control stick input?
     1. What level of confidentiality, or anonymity would require to have data collected from you in order to develop such a system.
     2. If this system required some personalization to each individual pilot in order to function well, would you be amenable to providing the system information (or allowing the system to collect the necessary information), such as your instrument-scanning patterns, or specific control strategies adopted for specific maneuvers?

APPENDIX C: Thematic Analaysis Code Descriptions

Table 9. Thematic analysis codes and descriptions: Gap I


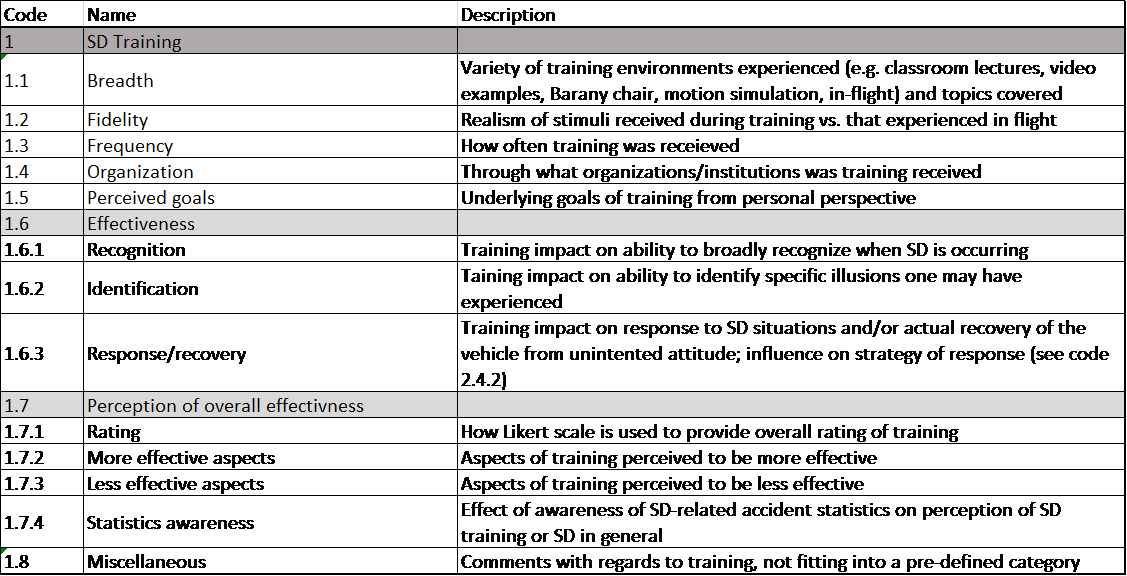


Table 10. Thematic analysis codes and descriptions: Gap II


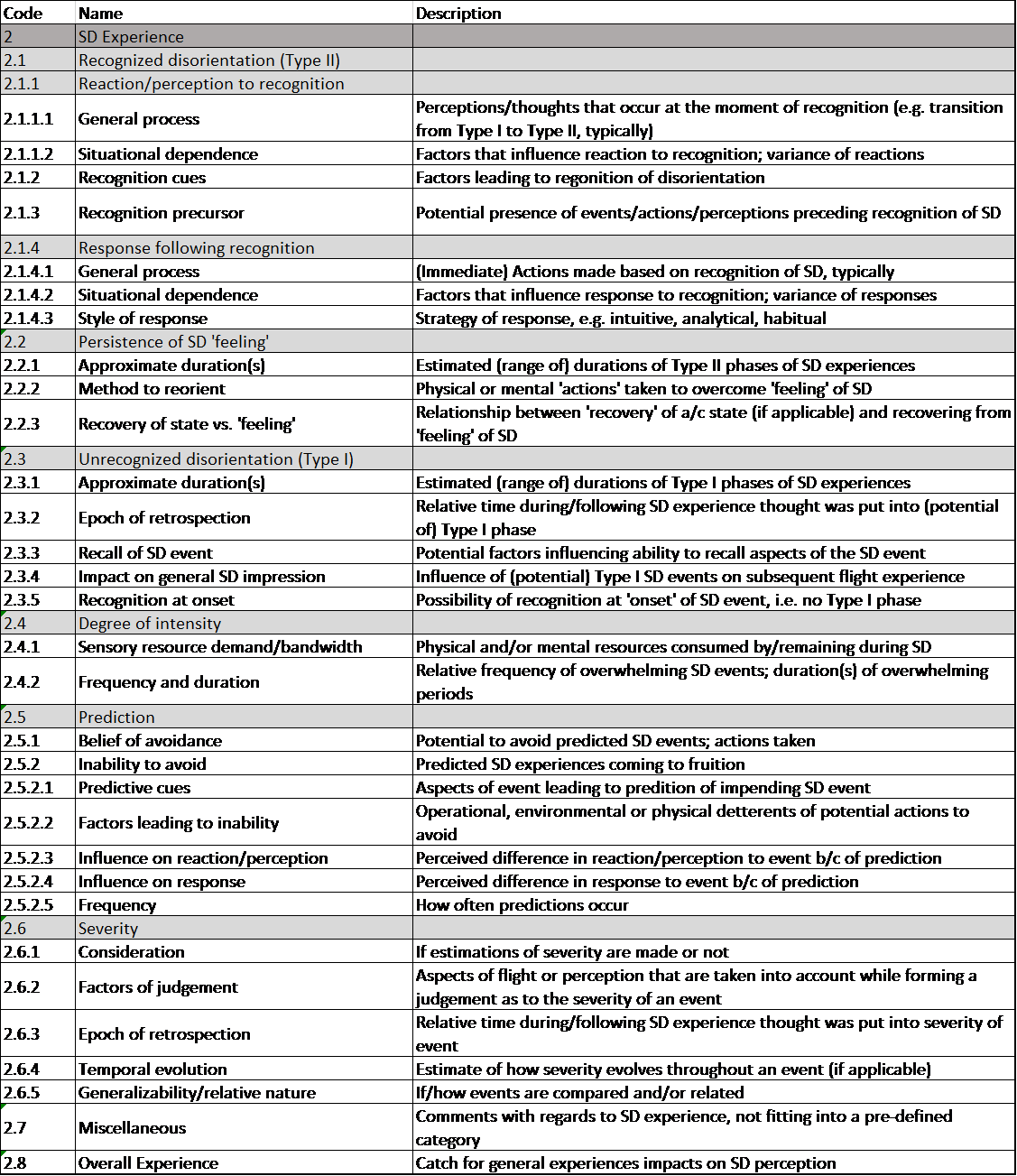


Table 11. Thematic analysis codes and descriptions: Gap III


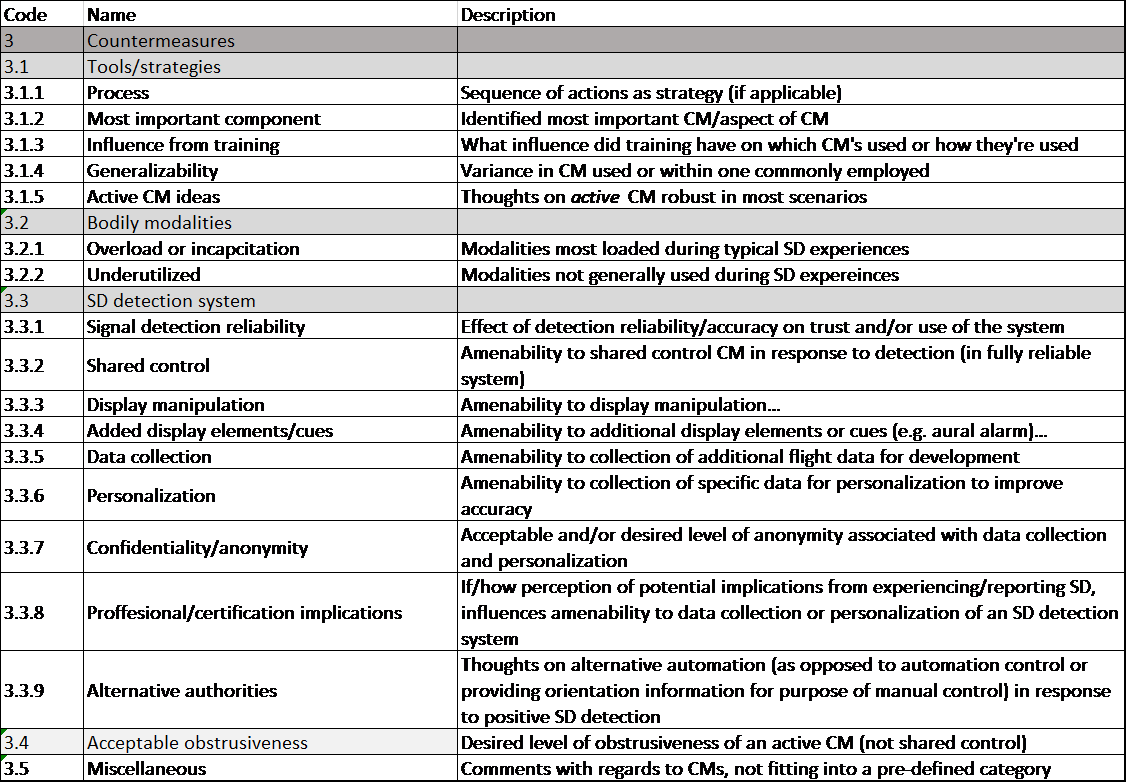

Supplement: Supplementary file 1 [file Supplementaryfile1.docx]
